# Hydrogen as a Co-electron Donor for Chain Elongation With Complex Communities

**DOI:** 10.3389/fbioe.2021.650631

**Published:** 2021-03-31

**Authors:** Flávio C. F. Baleeiro, Sabine Kleinsteuber, Heike Sträuber

**Affiliations:** ^1^Department of Environmental Microbiology, Helmholtz Centre for Environmental Research – UFZ, Leipzig, Germany; ^2^Technical Biology, Institute of Process Engineering in Life Science II, Karlsruhe Institute of Technology – KIT, Karlsruhe, Germany

**Keywords:** syngas fermentation, carboxylate platform, caproate, microbiome, microbial consortia, acidogenesis, methanogenesis, isobutyrate

## Abstract

Electron donor scarcity is seen as one of the major issues limiting economic production of medium-chain carboxylates from waste streams. Previous studies suggest that co-fermentation of hydrogen in microbial communities that realize chain elongation relieves this limitation. To better understand how hydrogen co-feeding can support chain elongation, we enriched three different microbial communities from anaerobic reactors (A, B, and C with ascending levels of diversity) for their ability to produce medium-chain carboxylates from conventional electron donors (lactate or ethanol) or from hydrogen. In the presence of abundant acetate and CO_2_, the effects of different abiotic parameters (pH values in acidic to neutral range, initial acetate concentration, and presence of chemical methanogenesis inhibitors) were tested along with the enrichment. The presence of hydrogen facilitated production of butyrate by all communities and improved production of *i-*butyrate and caproate by the two most diverse communities (B and C), accompanied by consumption of acetate, hydrogen, and lactate/ethanol (when available). Under optimal conditions, hydrogen increased the selectivity of conventional electron donors to caproate from 0.23 ± 0.01 mol e^–^/mol e^–^ to 0.67 ± 0.15 mol e^–^/mol e^–^ with a peak caproate concentration of 4.0 g L^–1^. As a trade-off, the best-performing communities also showed hydrogenotrophic methanogenesis activity by *Methanobacterium* even at high concentrations of undissociated acetic acid of 2.9 g L^–1^ and at low pH of 4.8. According to 16S rRNA amplicon sequencing, the suspected caproate producers were assigned to the family *Anaerovoracaceae* (*Peptostreptococcales*) and the genera *Megasphaera* (99.8% similarity to *M. elsdenii*), *Caproiciproducens*, and *Clostridium* sensu stricto 12 (97–100% similarity to *C. luticellarii*). Non-methanogenic hydrogen consumption correlated to the abundance of *Clostridium* sensu stricto 12 taxa (*p* < 0.01). If a robust methanogenesis inhibition strategy can be found, hydrogen co-feeding along with conventional electron donors can greatly improve selectivity to caproate in complex communities. The lessons learned can help design continuous hydrogen-aided chain elongation bioprocesses.

## Introduction

Ethanol, lactate, and sugars are conventional electron donors (EDs) that enable production of medium-chain carboxylates (MCC) through microbial chain elongation (CE) in anaerobic fermentation (Wu et al., 2019a). Despite the fact that EDs can be produced from low value lignocellulosic biomass, they are only formed as fast as hydrolysis rates of lignocellulose allow. As a consequence, low availability of EDs is a major bottleneck to achieve extractable quantities of MCC in bioreactors with lignocellulosic substrates. It is therefore not surprising that chain elongation studies have focused mostly on substrates that are less abundant, but rich in EDs such as corn silage (Lambrecht et al., 2019) and corn beer (Vasudevan et al., 2014; Urban et al., 2017) or other waste streams from alcoholic beverages industry (Kucek et al., 2016; Wu et al., 2018), food processing (Nzeteu et al., 2018; Contreras-Davila et al., 2020; De Groof et al., 2020), and dairy industry (Xu et al., 2018; Duber et al., 2020).

When ED concentration in the substrate does not suffice, many lab-scale studies opted in for supplementing chemical-grade lactate, ethanol or sugars during anaerobic fermentation to achieve high caproate productivities (Grootscholten et al., 2014; Roghair et al., 2018b). Moreover, yeast extract, commonly used as nutrient source and microbial growth enhancer (Grootscholten et al., 2013), should also be considered as a possible source of EDs since it is able to sustain some carboxylate production by itself (Richter et al., 2013; Chen et al., 2016; San-Valero et al., 2020). From an economic standpoint, supplementation of conventional EDs (yeast extract included) is merely a temporary solution. If MCC production through anaerobic fermentation is meant to become a more competitive and sustainable biorefinery process, it should not depend on costly ED supplementation (Richter et al., 2013; Chen et al., 2017).

Among the proposed solutions to overcome ED scarcity in MCC production, integration of CE with syngas fermentation has been proposed in several different configurations (Baleeiro et al., 2019), thus applying H_2_ and CO as alternative EDs for CE. Syngas (H_2_/CO_2_/CO) and water-gas shifted syngas (H_2_/CO_2_) can be produced by gasification of lignocellulosic biomass, making even the most recalcitrant fractions of lignocellulose bioavailable to anaerobic bacteria via the Wood-Ljungdahl pathway. Co-feeding H_2_ directly to the anaerobic microbiota is one of the simplest strategies to steer its electrons into acetate, ethanol, butyrate, and even caproate. Application of H_2_, CO_2_ (and occasionally CO) in fermentation systems can support MCC production through: (i) production of acetate, being the most common product of syngas fermentation (Infantes et al., 2020) and an electron acceptor in CE; (ii) production of ethanol, an ED for CE (Steinbusch et al., 2008); and (iii) direct production of MCC by species such as *Clostridium carboxidivorans* or *Eubacterium limosum* (Ramio-Pujol et al., 2015; Wade, 2015). Furthermore, H_2_ and CO_2_ may have either detrimental or beneficial thermodynamic and kinetic effects on carboxylate production depending on their partial pressure. For instance, abundant CO_2_ supply was reported to favor MCC production in mixed communities (Roghair et al., 2018a), but high partial pressure of H_2_ makes CE by *Clostridium kluyveri* less favorable (Schoberth and Gottschalk, 1969; Angenent et al., 2016). Besides, the continuous presence of H_2_ in the fermenter is a main factor that shapes the community composition (Wu et al., 2019b; Liu C. et al., 2020).

Although co-feeding of conventional EDs has been an extensively adopted strategy to increase MCC yields (Wu et al., 2019a; Stamatopoulou et al., 2020), only a handful of studies tested the effect of co-feeding H_2_ and CO_2_ together with conventional EDs, short-chain carboxylates (SCC), and/or complex substrates. Among these studies, some evidence has been shown for a net positive effect of H_2_ co-feeding (Steinbusch et al., 2011; Arslan et al., 2012; Nzeteu et al., 2018; Wu et al., 2019b; González-Tenorio et al., 2020; Liu C. et al., 2020). However, the underlying mechanisms and the involved microorganisms as well as the boundaries of such synergy remain unknown. In this study, we aimed to determine the effects of H_2_ feeding on MCC production by: (i) enriching three microbial communities from different inocula toward butyrate and MCC formation with H_2_ as ED; (ii) comparing the dynamics of the three microbial communities along the enrichment; (iii) identifying adequate fermentation parameters; and (iv) co-feeding conventional EDs (lactate and ethanol) and H_2_ in the last enrichment step.

## Materials and Methods

### Experimental Design of Batch Cultures

**FIGURE 1:**
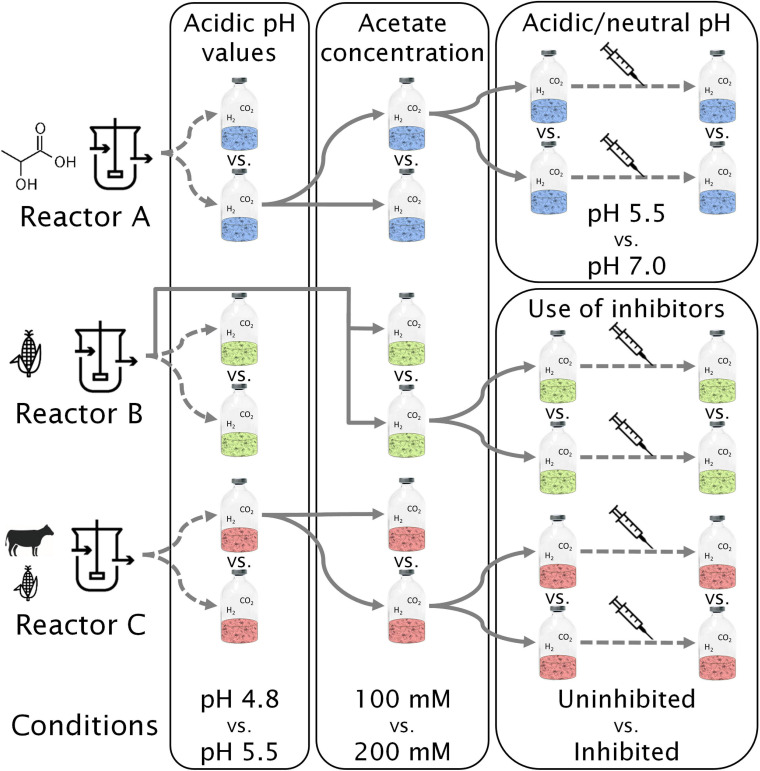
Enrichment scheme and overview of experiments. Reactor A was fed with lactate and xylan, reactor B with corn silage, and reactor C with corn silage and cow manure. The syringe icon symbolizes addition of conventional electron donors (lactate and ethanol). One bottle with H_2_/CO_2_ is depicted for each condition and experiment. Duplicates, H_2_-free, and abiotic controls are not shown. Full arrows mean transfer from selected bottles with 10% inoculation. Dashed arrows represent new experiments without dilution of the inoculum.

The experiments carried out along with the community enrichment are schematized in Figure 1. The duration of each experiment varied from 21 to 92 days, depending on the time to reach stable caproate concentration or complete acetate consumption (Supplementary Table 1). All conditions were tested in duplicates and included controls for the presence of H_2_ (using N_2_ instead) and abiotic controls (receiving 10% v/v of deionized, sterile water instead of inoculum). The batch experiments were done in 250 mL serum bottles filled with 50 mL culture liquid and capped with butyl rubber stoppers.

The microbial communities originated from three different types of anaerobic, mesophilic reactors operating at near neutral or slightly acidic pH. Community A stemmed from a continuous bench-scale acidogenic reactor operated for MCC production, fed with xylan and lactate, operating at pH 5.5, 38°C and a hydraulic retention time (HRT) of 8 days (Liu et al., 2020a). Community B stemmed from a continuous lab-scale acidogenic reactor operated for MCC production, fed with unsterile corn silage, equaling and inoculated from the reactor described by Lambrecht et al. (2019) (pH of 6.25, 38°C, HRT of 4 days). Community C stemmed from a full-scale anaerobic digester operated for biogas production, fed with cow manure and corn silage, operating at 37°C, neutral pH, and with an HRT of 42 days. The reactors from which communities A and B originated produced mainly acetate, butyrate, and caproate, whereas the reactor from which community C originated produced biogas (CH_4_ and CO_2_). Inocula from reactors B and C were sieved under N_2_ flow with a 0.355 mm mesh size to remove excessive amounts of solids.

Culture bottles were pressurized to approx. 2.0 bar_a_ of H_2_/CO_2_ mix (or N_2_/CO_2_ mix for H_2_-free controls) with 80 vol.% H_2_ (or N_2_) and 20 vol.% CO_2_ and incubated at 37°C in a rotatory shaker at 200 rpm. Headspaces were flushed and repressurized in the beginning of each experiment and whenever pressure was detected to be lower than 1.2 bar_a_. Before inoculation and (re)pressurization, the headspace of each bottle was flushed with 250 mL min^–1^ of gas mixture for 10 min to purge the previous headspace and to ensure anoxic conditions.

In the first batch experiment, communities were tested at pH values of 4.8 and 5.5 in their original reactor broths (Figure 1). Acetate (100 mM) was added to act as a buffer and to thermodynamically favor CE over the more common acetate formation from H_2_ and CO_2_. The conditions resulting in highest MCC production for the majority of the communities in each batch experiment were applied as baseline conditions in the next experiment. Thus, for testing the effect of acetate concentration (second batch experiment) and for the inhibition experiment (third batch experiment), an initial pH of 5.5 was set in all bottles. For the third experiment (effect of pH in community A and effect of methanogenesis inhibition in communities B and C), the initial acetate concentration was 200 mM. As part of the enrichment scheme, the best-performing carboxylate-producing pair of duplicates was selected to inoculate the succeeding batch culture.

The inhibition experiment aimed to investigate the competition between methanogenesis and MCC production through the use of chemical methanogenesis inhibitors. Since community A showed no methanogenic activity, the effects of pH values of 5.5 and 7.0 were compared instead of using methanogenesis inhibitors. The fourth experiment consisted in the addition of 60 mM lactate and 30 mM ethanol to the bottles of the third experiment (Figure 1), aiming to investigate the effect of H_2_ during lactate- and ethanol-based CE.

Starting from the second experiment, batch cultures were set up in a defined medium and inoculated with 10% reactor broth or broth from the first experiment (Figure 1). The basal growth medium was adapted from Liu et al. (2020a) differing in the absence of Na_2_CO_3_ and in the presence of 6.62 g L^–1^ sodium acetate and 1.16 g L^–1^ acetic acid (bottles with 100 mM acetate) or 13.24 g L^–1^ sodium acetate and 2.31 g L^–1^ acetic acid (bottles with 200 mM acetate). For the methanogenesis inhibition experiment, 10.5 g L^–1^ of sodium 2-bromo-ethanosulfonate (2-BES) was used for community C and 9 mL (ca. 4.5 kPa) of ethylene was added in the bottle headspace for community B. For 2-BES, the concentration used was based on Zinder et al. (1984). For ethylene, an amount just below the value tested on *Acetobacterium woodii* by Schink (1985) was used. For the addition of conventional EDs, 10 mL of pre-dissolved DL-lactic acid/ethanol in basal medium was added to each bottle to reach final concentrations of 5.4 g L^–1^ lactate (60 mM) and 1.4 g L^–1^ ethanol (30 mM) in order to start the experiment with 50 mL broth. During sampling procedures, the pH value was adjusted to 4.8, 5.5 or 7.0 with 4 M KOH or 4 M HCl if necessary. The main buffers in this study were acetate and other monocarboxylates (pK_a_∼4.8), the phosphate system (pK_a2_∼7.2), the carbonate system (apparent pK_a_∼6.1), and lactate (pK_a_∼3.8). Media were made anoxic by stirring them in an anaerobic glovebox for at least 3 h. Media were sterilized by autoclaving at 121°C for 20 min, except for vitamins and cysteine concentrates, which were sterilized by filtration with 0.2 μm cellulose acetate syringe filters (Labsolute, Germany).

### Analytical Methods

The serum bottles were monitored periodically for gas production and consumption, chemical composition of the gas and liquid phases, optical density at 600 nm (OD_600_), and community composition with sampling procedures adapted from Logroño et al. (2020). Carboxylates and alcohols in the liquid phase were measured with high performance liquid chromatography as described by Apelt (2020) using a modified method with a column temperature of 55°C and a flow rate of 0.7 mL min^–1^. Determination of gas composition was analyzed with gas chromatography with a thermal conductivity detector as described by Logroño et al. (2020). Monitoring of pressure and gas sampling in the headspace was done always before and after repressurization of bottles to determine the component balances in the gas phase.

### Microbial Community and Correlation Analyses

Amplicon sequencing of the 16S rRNA gene with Illumina MiSeq was done on cell pellets collected at the beginning and at the end of each batch. For community C in the first two experiments, genomic DNA was extracted from frozen cell pellets using the NucleoSpin Soil Kit (Macherey-Nagel, Germany) according to the manufacturer’s manual. For all other cell pellets, NucleoSpin Microbial DNA Kit (Macherey-Nagel, Germany) was used. Quality assessment, quantification, and storage of the extracted DNA as well as PCR and library preparation were done according to the protocols for 16S rRNA genes described by Logroño et al. (2020). Raw sequence data for this study was deposited at the EMBL European Nucleotide Archive (ENA) under the study accession number PRJEB40259^1^. The primers used are described by Klindworth et al. (2013) and target the V3 and V4 regions of the 16S rRNA gene. Primer sequences were removed from adapter-clipped reads using Cutadapt (Martin, 2011) and further sequence data analysis was done through the DADA2 workflow, using the amplicon sequence variant (ASV) approach as described by Callahan et al. (2016). According to read quality profiles, forward and reverse reads were truncated at the length of 278 and the other parameters of the workflow were used in their default values. Taxonomic assignments were done using the SILVA 138 reference database (Yilmaz et al., 2014; McLaren, 2020). Diversity analysis, filtering, agglomeration, normalization, and subsetting of the microbiome census data were realized with the phyloseq package for R (McMurdie and Holmes, 2013). All samples were rarified to an equal depth of 40,000 counts (lowest read number was 43,381). For the predominating ASVs, MegaBLAST (Morgulis et al., 2008) was used to find the most similar cultured species within the NCBI standard nucleotide collection and the 16S ribosomal RNA sequences database (NCBI, 2018). A Spearman correlation matrix was used with *p* < 0.01 for correlation analyses between ASV abundances and abiotic data considering the non-control culture bottles.

### Assumptions for Electron Balances, Rates, and Selectivity

To quantify the chemical fluxes in the cultures, electron balances were calculated. Electron balancing overcomes the errors that unmonitored H_2_O formation and consumption cause in mass and mole balances and gives a uniform basis to calculate the consumption and production of chemical compounds in terms of mol e^–^. To calculate biomass weight from biomass concentration (OD_600_), a factor of 0.456 g L^–1^ dry mass per OD_600_ unit was used, which is an average value for *Escherichia coli* cultures (Myers et al., 2013) and was confirmed in our laboratory to be a realistic in-between value for anaerobic cultures grown autotrophically (Logroño et al., 2020) and heterotrophically (Liu et al., 2020a) in similar media.

Supplementary Table 2 lists the components and their conversion factors considered in the balances. The relative standard deviations of the electron balances were typically between 2% and 20%, suggesting that the monitored compounds represent the bulk of the pool of electrons that were channeled during the fermentation. Error sources of the electron balances were most likely the decrease of broth volume due to sampling (typically 10% by the end of each batch), limited accuracy of the chemical analytics, and unmonitored compounds. As a convention, consumption of compounds is shown as negative values.

Production rates for the chemicals in the liquid and in the gaseous phase were calculated with Eqs (1) and (2), respectively.

(1) $$P\,r\,o\,d\,u\,c\,t\,i\,o\,n\,r\,a\,t\,e\,\left[m\,gL^{-1}\,d^{-1}\right]=\frac{C_{f}-C\,i\,\left[m\,gL^{-1}\right]}{d\,u\,r\,a\,t\,i\,o\,n\,o\,f\,e\,x\,p\,e\,r\,i\,m\,e\,n\,t\,\left[d\right]}$$

where *C_f* and *C_i* are the concentrations of the chemical at the end and beginning of the experiment, respectively,

(2) $$G\,a\,s\,p\,r\,o\,d\,u\,c\,t\,i\,o\,n\,r\,a\,t\,e\,\left[m\,gL^{-1}\,d^{-1}\right]=$$

$$\frac{m\left[mg\right]}{V_{broth}\left[L\right]\times \;duration\;of\;\exp eriment\left[d\right]}$$

where *V*_*broth*_ is the average volume of broth along the experiment and *m* is the accumulated gas mass produced in the period.

Selectivity of conventional EDs to caproate was calculated on the basis of electron equivalents according to Eq. (3).

(3) $$S\,e\,l\,e\,c\,t\,i\,v\,i\,t\,y\,\left[m\,o\,l\,e^{-}\,c\,a\,p\,r\,o\,a\,t\,e/m\,o\,l\,e^{-}\,E\,D\right]=$$

$$\frac{\Delta \,n_{c\,a\,p\,r\,o\,a\,t\,e}\,\left[m\,o\,l\,e^{-}\right]}{\Delta \,n_{e\,t\,h\,a\,n\,o\,l}+\Delta \,n_{l\,a\,c\,t\,a\,t\,e}+n_{Y\,E}\,\left[m\,o\,l\,e^{-}\right]}$$

where △*n*_*ethanol*_ and △*n*_*lactate*_ are the consumed ethanol and lactate, respectively, *n*_*YE*_ is the amount of yeast extract initially present in the medium, in this study 4.3 mmol e^–^ (500 mg L^–1^ in medium), and △*n*_*caproate*_ is the produced caproate. For the first experiment, which was done with the undiluted broth of reactors, an amount of yeast extract of 4.3 mmol e^–^ was assumed to account for the unmonitored substrates present in the broth.

## Results

Three different enrichment cultures A, B, and C were established. Along the enrichment, the effects of different pH values, acetate concentrations, and methanogenesis inhibition measures on their production performances and community developments were studied in consecutive batch experiments as shown in Figure 1. An overview of the butyrate and caproate production rates of the three communities at all conditions tested in this study is shown in Supplementary Table 3.

### Effect of Different Acidic pH Values

**FIGURE 2:**
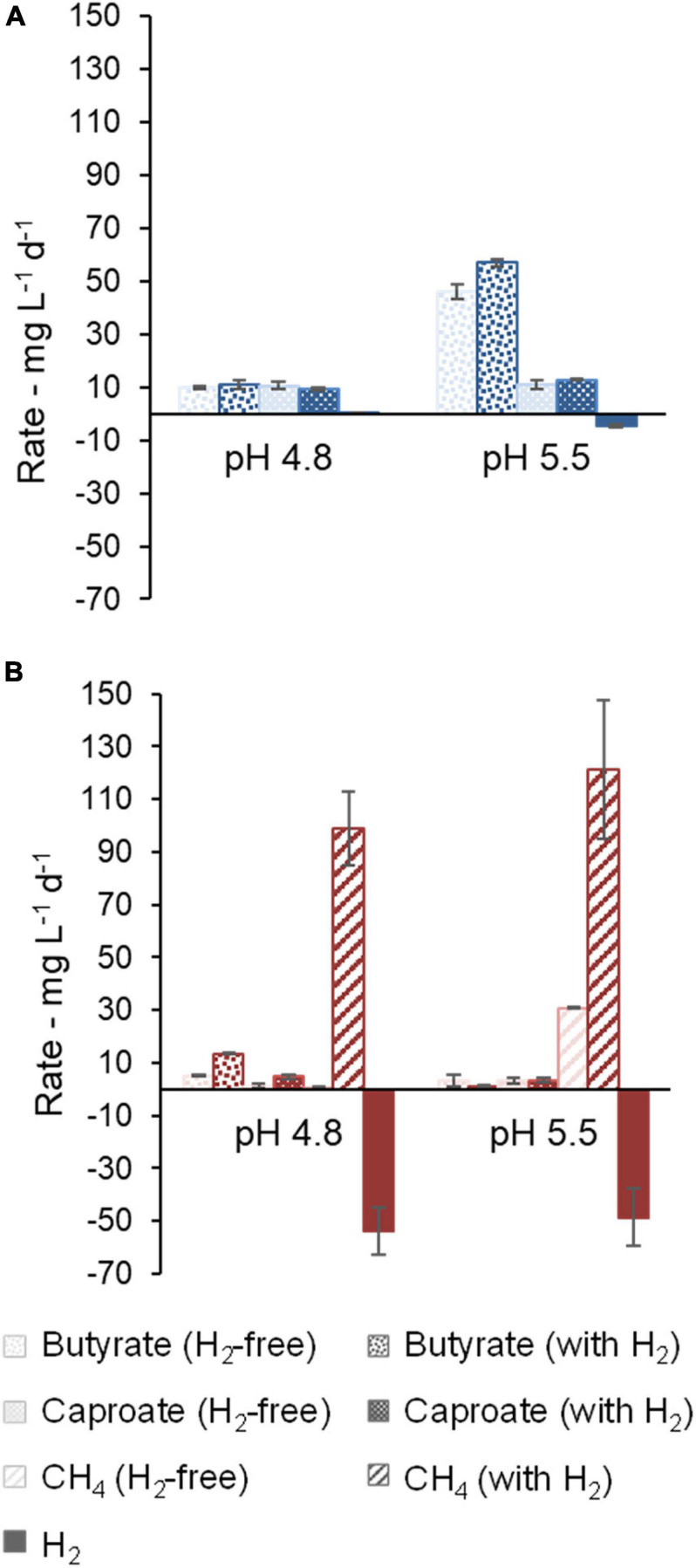
Effect of pH on the production rates (positive values) and consumption rates (negative values) of butyrate, caproate, CH_4_, and H_2_. Community A **(A)** and Community C **(B)** are shown. Results are not shown for community B, because it showed no activity after acidification to pH values of 4.8 and 5.5. Error bars represent standard errors.

First, the effect of H_2_ addition on the communities in their original broth at pH values of 5.5 and 4.8 was investigated and the cultures were compared with H_2_-free controls. Small H_2_ consumption and no methanogenic activity was detected in community A (Figure 2A). At pH 5.5, H_2_ addition resulted in the accumulation of on average 3712 mg L^–1^ (62 mM) more acetate (not shown) and 610 mg L^–1^ (6.9 mM) more butyrate in comparison to H_2_-free controls. Initially, broth from reactor A still contained substantial amounts of carboxylates as well as unconsumed xylan and lactate as substrates, which contributed to some caproate production regardless of pH or H_2_ addition. H_2_ addition caused no significant difference in caproate production rates in community A at pH 4.8 and pH 5.5. However, the community showed higher H_2_ consumption rates at pH 5.5 (Figure 2A).

By monitoring of pressure and composition of gas and liquid chemicals, no microbial activity was detected in the broth from reactor B after acidification to pH 5.5 or 4.8. Broth of reactor B originally contained the highest concentration of carboxylates among the three broths used in this study (data not shown).

In comparison to community A, community C consumed more H_2_ (Figure 2B). CH_4_ was the main product, even at initial pH values as low as 4.8 and 5.5. At pH 5.5, acetate (100 mM) was depleted relatively fast at rates of 474 ± 2 mg L^–1^ d^–1^ (without H_2_) and 278 ± 50 mg L^–1^ d^–1^ (with H_2_). Acetate consumption caused the loss of buffering capacity and consequently a tendency for pH increase toward neutral conditions. At pH 4.8 with H_2_, slow acetate consumption occurred at a rate of 35 ± 3 mg L^–1^ d^–1^, but no acetate consumption was observed in H_2_-free controls (−0.9 ± 1.8 mg L^–1^ d^–1^). In this experiment with community C, accumulation of MCC was outperformed by methanogenic activity (Figure 2B). Still, at pH 4.8, a higher caproate concentration of 360 ± 28 mg L^–1^ (H_2_-free: 50 ± 38 mg L^–1^) was detected in the presence of H_2_, whereas at pH 5.5 no such difference was seen (131 ± 13 mg L^–1^ and 124 ± 3 mg L^–1^ in H_2_-containing and H_2_-free bottles, respectively).

### Effect of Acetate Concentration

Since higher concentrations of acetate are known to selectively favor acidogens and CE over methanogens (Zhang et al., 2018; Cavalcante et al., 2020), the performances of cultures at acetate concentrations of 200 mM and 100 mM were compared in the following enrichment step.

**FIGURE 3:**
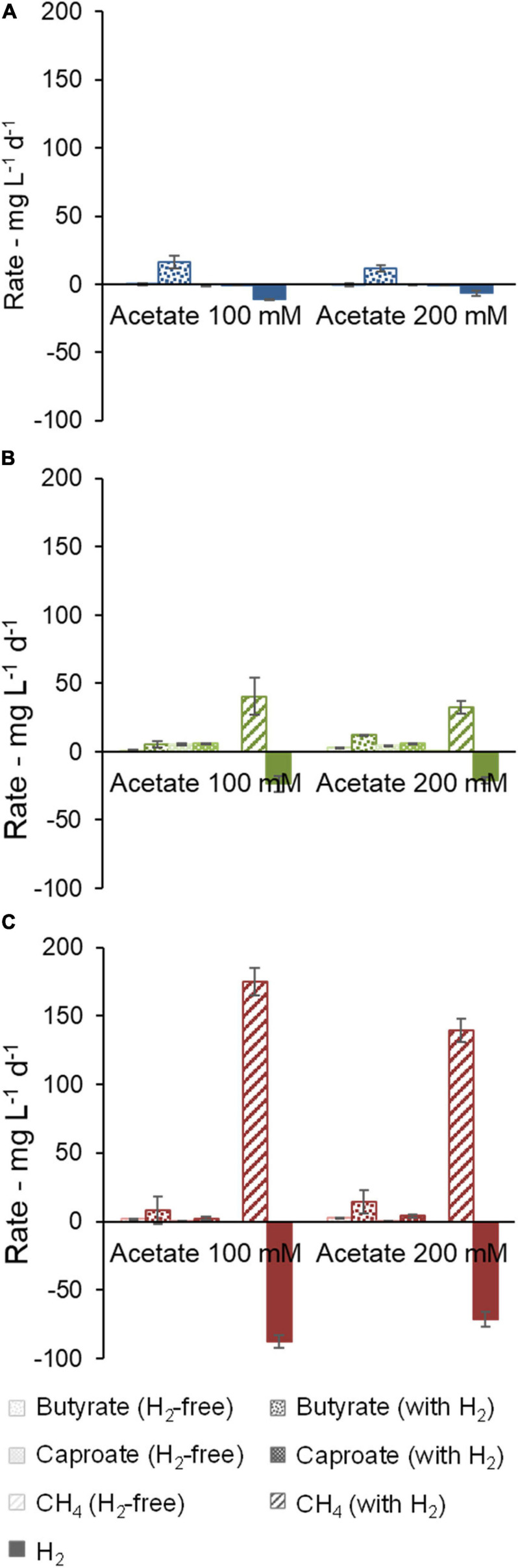
Effect of acetate concentration on production and consumption rates of butyrate, caproate, CH_4_, and H_2_. Community A **(A)**, Community B **(B)**, and Community C **(C)** are shown.

Figure 3 shows production and consumption rates for caproate, CH_4_, and H_2_. For a deeper analysis including acetate and *i*-butyrate, Supplementary Figure 1 presents balances in terms of electron equivalents. In Figure 3A, it can be seen that community A, free of methanogenic activity, could not produce caproate and its H_2_ consumption (10.56 ± 0.01 mg L^–1^ d^–1^ and 6.5 ± 1.9 mg L^–1^ d^–1^ at 100 mM and 200 mM acetate, respectively) coincided with an accumulation of butyrate (between 12 ± 2 mg L^–1^ d^–1^ and 16 ± 5 mg L^–1^ d^–1^ at 200 mM and 100 mM, respectively). Higher acetate concentration was detrimental to H_2_ consumption and butyrate production of this community, indicating that the microbiota may have been affected by acid inhibition.

Acetate concentration did not have a strong influence on carboxylate production by community B, whereas H_2_ presence increased butyrate production slightly (Figure 3B). Communities B and C showed hydrogenotrophic methanogenic activity, which was partially suppressed by a higher acetate concentration (Figures 3B,C). There were no signs of methane production associated with acetate consumption in any of the cases. Accumulation of caproate, butyrate, and *i*-butyrate by community C was more favorable at higher acetate concentrations and in H_2_-containing bottles (3.1 ± 0.7 mmol e^–^, 9.1 ± 2.7 mmol e^–^, and 3.1 ± 1.9 mmol e^–^, respectively), even though such effect was dwarfed by 209 ± 12 mmol e^–^ CH_4_ produced by methanogenic activity (Figure 3C and Supplementary Figure 1C).

As methanogenesis impeded enrichment of acidogenic microorganisms in communities B and C even with 200 mM acetate, the effect of methanogenesis inhibition was tested on these communities.

### Effect of Acidic/Neutral pH (Community A)

In the following enrichment step, pH values of 5.5 and 7.0 were tested on community A (Figure 1). Neither production of caproate nor consumption of H_2_ in significant amounts were observed at this step (Supplementary Figure 2A). Community A produced only butyrate (19 ± 9 mg L^–1^ d^–1^) in the presence of H_2_ and at pH 5.5. Subsequently, effects of pH values of 5.5 and 7.0 (and presence of H_2_) were further compared after the addition of lactate (5400 mg L^–1^) and ethanol (1380 mg L^–1^) to the bottles with community A (Figure 1). Regardless of the pH value or H_2_ presence, community A stopped consuming ethanol at a concentration of 185 ± 28 mg L^–1^ within the first five days, whereas lactate consumption halted at a concentration of 448 ± 181 mg L^–1^ after nine to 19 days. Lactate and ethanol addition allowed higher butyrate production rates of 317 ± 107 mg L^–1^ d^–1^ (pH 5.5, with H_2_) and 172.9 ± 0.7 mg L^–1^ d^–1^ (pH 7.0, with H_2_) in comparison to previous batches (Supplementary Figure 2B). Some H_2_ consumption could be maintained at pH 5.5 (14 ± 10 mg L^–1^ d^–1^) and was more likely connected to butyrate production (Supplementary Figure 3A). Nearly no caproate production could be maintained by community A at any of the pH values. At pH 7.0 in the absence of H_2_, probably some lactate was converted to propionate (Supplementary Figure 3A).

### Effect of Methanogenesis Inhibition (Community B and Community C)

**FIGURE 4:**
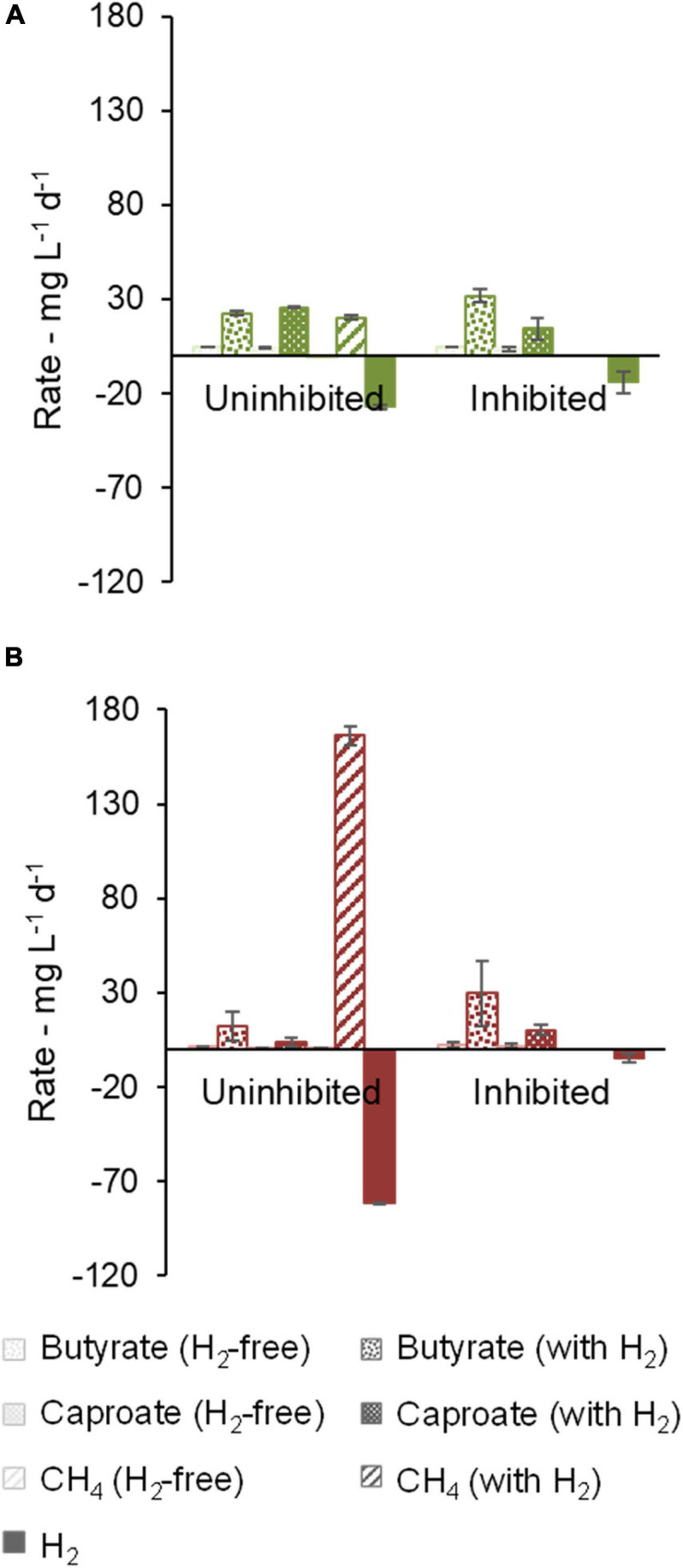
Effect of methanogenesis inhibition on production and consumption rates of butyrate, caproate, CH_4_, and H_2_. Community B **(A)** and Community C **(B)** are shown.

For communities B and C, methanogenic activity was suppressed by the use of chemical inhibitors (Figure 4). H_2_ consumption of 14.1 ± 5.6 mg L^–1^ d^–1^ (community B) and 4.4 ± 1.6 mg L^–1^ d^–1^ (community C) was observed in the cultures with inhibitor, but it was smaller than in the cultures without inhibitor. In this enrichment step, community B proved to be a better H_2_ consumer than community C when methanogenesis was inhibited. Moreover, butyrate and caproate production rates of community B were clearly higher when H_2_ was present. Caproate concentrations peaked at 1.9 g L^–1^ after 39 days under uninhibited conditions but averaged to 1.23 ± 0.03 g L^–1^ at the end of the experiment (48 days), suggesting that some caproate may have been consumed. Caprylate, which can be formed by CE coupled to caproate consumption, was not detected. Significant accumulation of *i*-butyrate was also observed in both communities in bottles with H_2_. At the end of the experiment with community B, *i*-butyrate concentrations were 1.5 ± 0.1 g L^–1^ and 1.75 ± 0.06 g L^–1^ under uninhibited and inhibited conditions, respectively.

Regarding community C, the highest butyrate and caproate production rates were observed when H_2_ and the methanogenesis inhibitor were present (Figure 4B). In this case, the inhibitor was decisive to prevent routing of H_2_ to methane and thus to facilitate carboxylate production. Supplementary Figure 4 illustrates how the methanogenesis inhibitor could avoid the periodical H_2_ depletion in community C cultures.

To test communities B and C for the effects of H_2_ addition and methanogenesis inhibition in the presence of lactate and ethanol, 5400 mg L^–1^ lactate and 1380 mg L^–1^ ethanol were added to the culture bottles (Figure 1).

**FIGURE 5:**
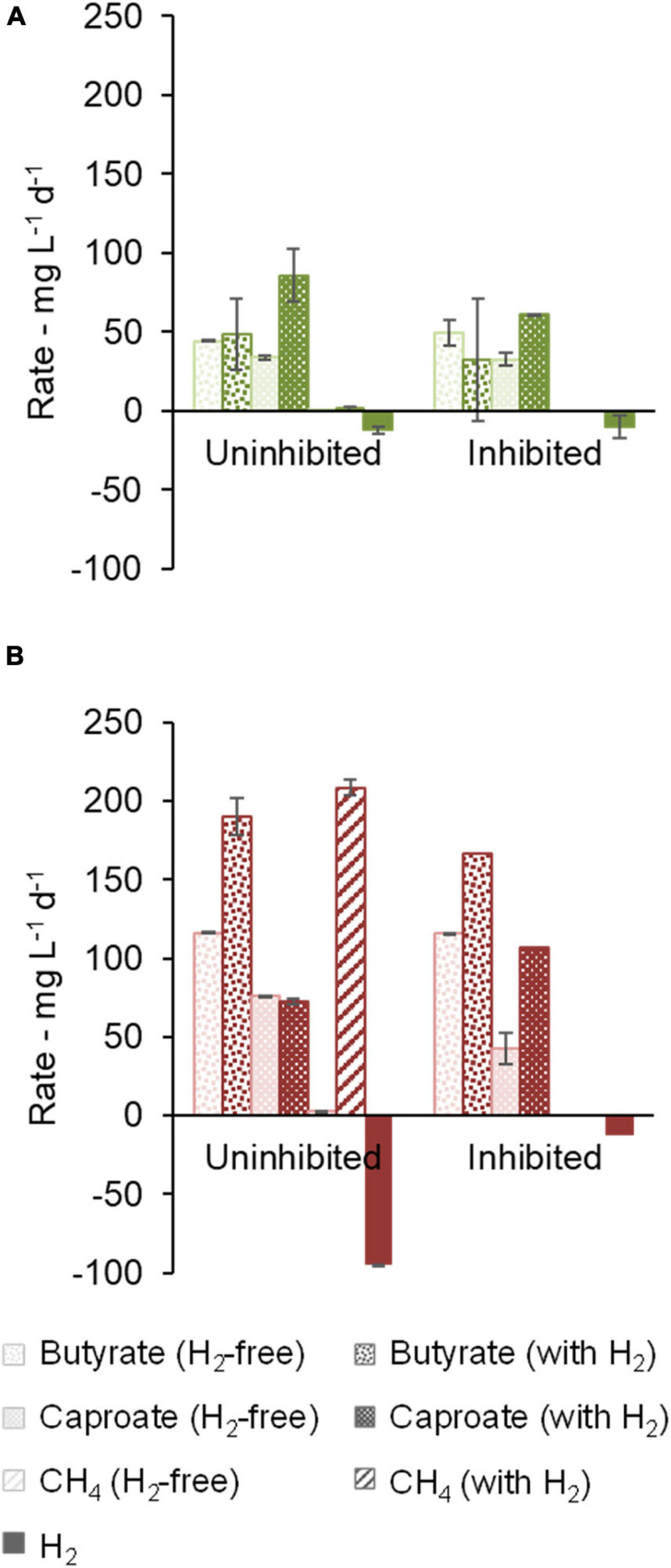
Effect of methanogenesis inhibition in the presence of lactate and ethanol on production and consumption rates of butyrate, caproate, CH_4_, and H_2_. Community B **(A)** and Community C **(B)** are shown. One of the duplicates of community C with H_2_ and inhibitor did not grow and no standard error bar is shown for this condition.

Cultures of community B consumed lactate completely within the first 12 days whereas ethanol was completely consumed within 19 to 26 days. Most of the H_2_ consumption occurred in the same period as lactate and ethanol consumption (Supplementary Figure 5). Lactate and ethanol addition allowed much higher production rates of carboxylates in comparison to the previous experiments, up to 86 ± 17 mg L^–1^ d^–1^ caproate in community B (Figure 5A) and 190 ± 12 mg L^–1^ d^–1^ butyrate in community C (Figure 5B). Methanogenesis nearly stopped in community B without the use of an inhibitor (1 ± 2 mg L^–1^ d^–1^ CH_4_) whereas it remained active in community C when an inhibitor was not present (209 ± 39 mg L^–1^ d^–1^ CH_4_). In the presence of the conventional EDs, non-methanogenic H_2_ consumption was up to 12 ± 2 mg L^–1^ d^–1^ in community B (uninhibited conditions) and 13 mg L^–1^ d^–1^ in the bottle with inhibitor in community C (no standard error available).

Community B channeled between 79% and 143% more electrons into caproate when H_2_ was present, as shown in the electron balance in Supplementary Figure 3B. Among the bottles with H_2_, bottles with inhibitor accumulated somewhat less caproate, but more *i*-butyrate. The positive effect of H_2_ on the caproate production by community C was more distinct in the presence of the methanogenesis inhibitor, which guaranteed H_2_ availability along the whole fermentation, similarly, to what was observed before lactate and ethanol addition. The main end-products in terms of electron equivalents were (in descending concentrations) caproate, *i*-butyrate, and butyrate in community B (Supplementary Figure 3B) and butyrate, caproate, and *i*-butyrate in community C (Supplementary Figure 3C).

### Development of Caproate Production and Selectivity Along the Enrichment Experiments

**FIGURE 6:**
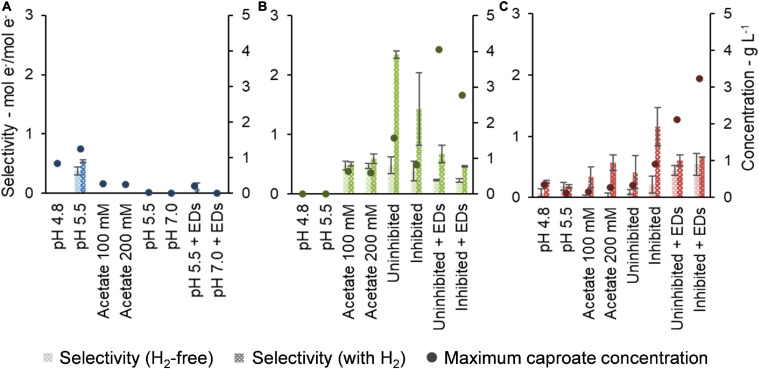
Selectivity of conventional electron donors (lactate, ethanol, and yeast extract) to caproate and maximum caproate concentration achieved by Community A **(A)**, Community B **(B)**, and Community C **(C)**.

To show the impact of H_2_ presence and of each condition on the efficiency of caproate production, Figure 6 depicts the selectivity to caproate. Additionally, Figure 6 also presents the performance of each community in terms of highest caproate concentration achieved for the tested condition.

During the enrichment, maximum caproate concentrations increased with communities B and C, whereas community A lost the ability to produce caproate (Figure 6). All maxima of caproate concentration were achieved under the presence of H_2_. Communities B and C both achieved peak concentrations after lactate and ethanol were added but methanogenesis inhibition had different effects on them. Community B peaked at 4.0 g L^–1^ in the absence of the inhibitor, while community C peaked at 3.2 g L^–1^ in the presence of the inhibitor. Community A achieved a maximum concentration of 1.2 g L^–1^ in the first experiment at pH 5.5, probably due to residual substrates in the original reactor broth. Nevertheless, H_2_ presence favored production of butyrate by this community in the latest experiment at both pH values of 5.5 and 7.0 (Supplementary Figure 3A).

Selectivities of conventional EDs, i.e., lactate, ethanol, and yeast extract, to caproate in the presence of H_2_ were at least as high as under H_2_-free conditions (Figure 6). In the later steps of the enrichment, selectivities of communities B and C were more clearly enhanced by the presence of H_2_. The highest selectivity achieved by community B was 2.34 ± 0.06 mol e^–^/mol e^–^ under uninhibited conditions, while community C achieved 1.2 ± 0.3 mol e^–^/mol e^–^ when methanogenesis was inhibited. Before the addition of lactate/ethanol, the conventional EDs available were yeast extract (500 mg L^–1^, 86 mmol e^–^ L^–1^) and residual substrates from the original reactor broth. Therefore, a drop in selectivity was observed when lactate (60 mM, 720 mmol e^–^ L^–1^) and ethanol (30 mM, 360 mmol e^–^ L^–1^) were added in higher concentrations.

Overall, no ethanol accumulation was observed and only trace amounts of valerate, *i*-valerate, *i*-caproate, and caprylate were detected along the study. Abiotic controls showed no activity, except for one control bottle for community A at pH 7.0. This abiotic control bottle was contaminated during the experimental procedure for the addition of lactate and ethanol and therefore not considered further.

### Community Structure

**FIGURE 7:**
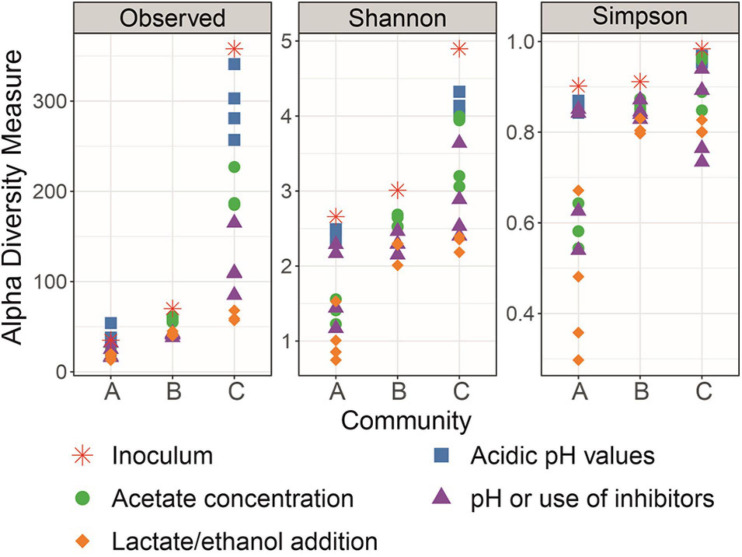
Diversity of Community A, Community B, and Community C throughout the enrichment in terms of richness (observed ASVs), Shannon index, and Simpson index.

Amplicon sequencing revealed different degrees of diversity and community composition of the original inocula for enrichment of the communities A, B, and C. As shown in terms of richness in Figure 7, inoculum diversity of community C was the highest, followed by the inocula of communities B and A, respectively. Shannon and Simpson indices are also presented in Figure 7 to help visualize the decrease in structural complexity of each community along the enrichment. At the end of the enrichment, communities B and C presented Shannon and Simpson indices of the same order of magnitude, whereas the diversity of community A was characterized by lower values.

**FIGURE 8:**
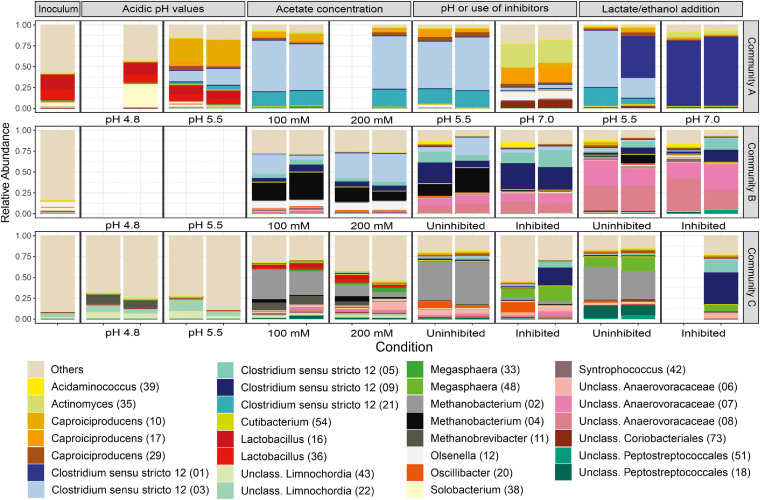
Community profiles resolved to the ASV level (ASV numbers in parentheses) at each condition tested with H_2_. The 30 most abundant ASVs in the dataset are shown. Duplicates are shown. Slots left blank represent samples that could not be sequenced.

By following the community development from the inoculum until the end of the lactate/ethanol addition experiment, Figure 8 shows the community compositions of the cultures with H_2_. Additionally, community compositions of all cultures including H_2_-free controls can be found in Supplementary Figure 6. Despite the fact that all communities comprised the genera *Caproiciproducens* and *Clostridium* sensu stricto 12 at the end of the enrichment, there were fundamental differences on the ASV level within these genera (Figure 8). Supplementary Table 4 provides the taxonomic affiliation for the most abundant ASVs found in this study.

Community A, initially dominated by *Caproiciproducens* ASV 10 at pH 5.5, shifted to the dominance of three ASVs assigned to *Clostridium* sensu stricto 12 (ASVs 1, 3, and 21) and of *Caproiciproducens* ASV 17 (Figure 8). Community B developed to a more diverse consortium composed mainly of two ASVs assigned to the family *Anaerovoracaceae* (ASVs 7 and 8), two ASVs assigned to *Clostridium* sensu stricto 12 (ASVs 5 and 9), *Caproiciproducens* ASV 29, and *Methanobacterium* ASV 4, which persisted in one of the uninhibited duplicates (Figure 8). Community C was finally composed of *Clostridium* sensu stricto 12 ASVs 5 and 9, *Anaerovoracaceae* ASV 6, *Megasphaera* ASV 48, and *Caproiciproducens* ASVs 10 and 29 in the culture with H_2_ and inhibitor (Figure 8). The presence of the methanogenesis inhibitor shaped community C more strongly than community B. In the absence of the inhibitor, *Methanobacterium* ASV 2 was dominant in community C. Besides *Megasphaera* ASV 48, *Peptostreptococcales-Tissierellales* ASV 18 was the most enriched bacterial taxon in the absence of the inhibitor (Figure 8).

The most enriched *Clostridium* spp. could be divided into two groups: ASV 1 with 100% BLAST identity to *Clostridium tyrobutyricum* and ASVs 3, 5, 9, and 21 with high BLAST similarity (>97%) to *Clostridium luticellarii* (Supplementary Table 4). *Clostridium* sensu stricto 12 (ASVs 3, 5, 9, and 21) were the most enriched *Clostridium* spp. and two of them (ASVs 3 and 21) were positively correlated to hydrogen consumption in community A (correlation coefficients of 0.77 and 0.84, respectively) (Supplementary Figure 7). The *C. luticellarii*-related ASVs 5 and 9 also correlated positively to some indicators of caproate formation in communities B and C. ASV 9 correlated to selectivity to caproate in community B (coefficient of 0.73, Supplementary Figure 8) and ASVs 5 and 9 to maximum caproate concentration in community C (0.70 and 0.67, respectively, Supplementary Figure 9).

The most abundant ASV of the genus *Caproiciproducens*, ASV 17, found in the latter enrichment phase of community A, had 100% BLAST identity with strain BL-6 (Liu et al., 2020b), a strain isolated from the same bioreactor that community A stemmed from. *Caproiciproducens* ASV 17 was different from the *Caproiciproducens* ASVs originally present in community A and from those present in communities B and C (ASVs 10 and 29). *Caproiciproducens* ASV 10 correlated to maximum caproate concentration in community A (0.55, Supplementary Figure 8) and community C (0.67), while *Caproiciproducens* ASV 29 correlated to caproate formation rate in community C (0.41, Supplementary Figure 9). ASVs 10 and 29 were distantly related to *Caproicibacter fermentans* (95% similarity, Supplementary Table 4).

Among the *Anaerovoracaceae*, ASVs 7 and 8 were enriched in community B and correlated to the butyrate production rate (0.85 and 0.83, respectively). ASV 8 correlated to the caproate production rate (0.91) and selectivity (0.80, Supplementary Figure 8). *Anaerovoracaceae* ASV 6 was present in community C, however, no significant correlation could be found (Supplementary Figure 9).

*Megasphaera* ASV 48 enriched in community C only correlated to caproate selectivity and caproate maximum concentration at a higher significance level of *p* < 0.05 (data not shown) but not at *p* < 0.01 (Supplementary Figure 9). *Megasphaera* ASV 48 had a BLAST similarity of 99.8% to *Megasphaera elsdenii* (Supplementary Table 4), a well-known caproate producer.

*Methanobacterium* ASVs 2 and 4 were responsible for methane formation in communities B (ASV 4 with correlation factor of 0.92, Supplementary Figure 8) and C (ASV 2 with correlation factor of 0.60, Supplementary Figure 9) under uninhibited conditions (Figure 8).

## Discussion

The enrichment experiments allow discussion from three perspectives: (i) strategies in dealing with methanogenesis, which revealed to be the main competitive pathway; (ii) the potential to improve caproate production with added H_2_; and (iii) identification of the key microbial players that emerged during the enrichment.

### The Methanogenesis Factor

Coincidently, the communities that could produce caproate in the presence of H_2_ were the same as those that presented hydrogenotrophic methanogenesis. In community C, a pH value of 4.8 was enough to inhibit acetoclastic methanogenesis. On the other hand, hydrogenotrophic methanogenesis was still observed at pH 4.8 (with 100 mM acetate) with an activity slightly lower than at pH 5.5 (Figure 2B), suggesting a relevant, yet surmountable acid inhibition of methanogens. Considering the fact that methanogenic activity has been rarely reported at such low pH (Savant et al., 2002; Horn et al., 2003), the methane formation observed in the first experiment with community C could be due to the high solids content of the biogas reactor broth, which can shelter microorganisms through mass transfer gradients (Abbassi-Guendouz et al., 2012). Surprisingly, community C continued to present a robust methanogenic activity, which channeled most of the H_2_ to CH_4_ even at strongly inhibiting pH (5.5) and acetate concentration (200 mM) conditions (Zhang et al., 2018) in the following enrichment step (Figure 3). When not actively suppressed by a chemical inhibitor, hydrogenotrophic methanogenesis was recurrent in community C throughout all the experiments, while methanogens faded out from community B along the enrichment without the need for an inhibitor (Figures 3–5). Differently, acetoclastic methanogenesis played only a minor role at pH 5.5 and 200 mM acetate, as suggested by the small consumption of acetate in the electron balances for community C (Supplementary Figures 1C, 4C), where CH_4_ production was only slightly higher than H_2_ consumption.

While the use of a methanogenesis inhibitor on community C (2-BES) increased production rates and selectivity to caproate (Figures 4B, 5C), the inhibitor used on community B (ethylene) had an opposite effect (Figures 4A, 5A, 6B). The inhibition mechanism of ethylene on methanogens has not been clearly elucidated, however, it is expected to be different from that of 2-BES (Schink, 1985; Liu et al., 2011). To the best of our knowledge, no negative effects of ethylene on acetogens have been reported so far. In the absence of inhibitors, methanogenic activity was much stronger in community C than in community B to a point where H_2_ became limiting in community C but not in community B. If 2-BES had detrimental effects on community C, these would have been shadowed by the effects of higher H_2_ availability (Supplementary Figure 4). Another possibility is that ethylene does not inhibit *i*-butyrate production whereas 2-BES is reported to do so (Huang et al., 2020). In fact, just as much electrons were channeled to *i*-butyrate (12 ± 4 mmol e^–^) as to caproate (12.2 ± 0.2 mmol e^–^) when ethylene was used (Supplementary Figure 3B), whereas less electrons were channeled to *i*-butyrate (26 mmol e^–^) than to caproate (36 mmol e^–^) when 2-BES was used (Supplementary Figure 3C). Although *i*-butyrate can theoretically be isomerized back to butyrate (Tholozan et al., 1988), *i*-butyrate production may compete with caproate formation when butyrate is the electron acceptor. The inhibition of 2-BES on *i*-butyrate formation was previously reported, but its mechanism is still not clear (Angelidaki and Ahring, 1995; Huang et al., 2020). According to the results found here, inhibition of *i*-butyrate formation by 2-BES may also inflate MCC yields. Thus, for more conservative estimates of MCC formation in systems with high potential to produce branched-chain carboxylates, we suggest the use of ethylene as methanogenesis inhibitor instead of 2-BES.

High salinity may also have inhibited methanogens, in particular in the later stages of the enrichment when acetate concentration was doubled and 2-BES was used. In this study, medium salinity started at around 14 g L^–1^ (NaCl equivalents, in experiments with 100 mM acetate), increased to 17 g L^–1^ in cultures with 200 mM acetate, and finally increased up to 22 g L^–1^ when 2-BES was added in cultures of community C. Notably, De Vrieze et al. (2016) reported a shift from methanogenesis to carboxylate production in upflow anaerobic sludge blanket reactors at salinity values higher than 19 g L^–1^ (calculated from conductivity value according to McDougall and Barker (2011)). Considering that no controls for salinity were used, the extent to which salinity inhibited methanogens in this study is unclear. Methanogens remained active in inhibitor-free cultures of community C at 200 mM acetate and about 17 g L^–1^ NaCl equivalents (Figures 4B, 5B). At the highest salinity values (22 g L^–1^), 2-BES was present and, as expected (Zinder et al., 1984), completely inhibited methanogens.

### Enhancing Caproate Production

Although the production of caproate with H_2_ as main ED could be increased in most communities, the maximum caproate concentrations of 1.5 g L^–1^ still fell short of the values around 10 g L^–1^ reached by the best-performing anaerobic fermenters fed with conventional EDs (De Groof et al., 2019). Besides, when only H_2_ (besides 500 mg L^–1^ yeast extract) was the available electron donor, small caproate production rates up to 25.7 ± 0.7 mg L^–1^ d^–1^ were observed (Figure 4A), which is in the same order of magnitude as observed by Zhang et al. (2013). Hence, to benefit from the effects of H_2_ on MCC production, two conventional EDs (lactate and ethanol) were co-fed with H_2_, thereby increasing production rates and concentrations of caproate in communities B and C. In the presence of lactate and ethanol, caproate formation rates increased to 107 mg L^–1^ d^–1^ (Figure 5). We expect the maximum caproate concentration (4.0 g L^–1^) and production rate (107 mg L^–1^ d^–1^) to have plenty of room for improvement as many important parameters (e.g., substrate ratios, pH, and temperature) have not been optimized in this study. For instance, assuming an electron donor to electron acceptor ratio of 1:1, the concentrations of ethanol and lactate used in this study could sustain up to 6.5 g L^–1^ caproate. In comparison to the anaerobic fermentation of lactate in the absence of H_2_, the observed benefits of H_2_ addition were higher concentrations, selectivities, and production rates of both caproate and butyrate. Regarding the preferred fermentation strategies for the concept, those that can maintain a high concentration of acetate in the bioreactor (e.g., in-line extraction of MCC, operation at lower pH values, inhibition of methanogenesis) can be useful to improve MCC production despite the known inhibiting effects of acetate on microbial communities (Agler et al., 2014; Zhang et al., 2018). It is assumed that an acetate concentration of 200 mM, such as in this study, is an attainable condition in anaerobic fermenters fed with lignocellulosic biomass, especially when coupled with in-line extraction for MCC, such as liquid-liquid extraction (Agler et al., 2014; Kaur et al., 2020). As a next step, the concept of H_2_ and conventional EDs co-feeding can be adapted to continuous reactors to test its feasibility under conditions closer to an industrial-scale fermenter. During the upscaling process, lessons learned from syngas fermentation research can be useful to tackle the challenges of feeding H_2_ as a substrate, such as micro-sparging to overcome low gas-mass transfer rates and using bubble-column reactors to keep power input low (Takors et al., 2018). Moreover, at industrial scale, renewable H_2_ is ideally sourced from water hydrolysis fueled by renewable energy or from lignocellulose gasification (Baleeiro et al., 2019).

Under conditions tested here, H_2_ addition could not sustain production of medium-chain alcohols. Even though the slightly acidic pH values used in most of the experiments are known to favor production of solvents such as butanol and hexanol (González-Cabaleiro et al., 2013; Ganigue et al., 2016), none of these alcohols was detected in this study. Based on the current knowledge on homoacetogenic pure cultures, when solvents are the desired products, co-feeding with CO-containing gases, such as syngas, is suggested (Diender et al., 2019; Infantes-López, 2020).

### Key Players in H_2_-Aided Chain Elongation

Community A, in contrast to the more diverse communities B and C, did not show the ability to produce caproate when only H_2_ and yeast extract were the available EDs. One reason could be a missing link in the H_2_-to-caproate metabolic chain, i.e., the Wood-Ljungdahl pathway, solventogenesis, interspecies ED transfer or reverse β-oxidation pathway. Considering that only a handful of cultured bacterial species possessing more than one of these capabilities are known (Angenent et al., 2016; Bengelsdorf et al., 2018), a high diversity inoculum improves the chances to obtain an acidogenic community that can profit from H_2_ as a co-electron donor. Nevertheless, after the community is enriched for H_2_-aided CE, high microbial diversity may not be a requirement for the bioreactor to work optimally.

Based on the correlations with caproate production and selectivity (Supplementary Figures 7–9), four taxonomic groups that might be involved in the caproate metabolism were identified: members of the genus *Clostridium* sensu stricto 12 (ASVs 3, 5, 9, and 21) related to *C. luticellarii*, members of the family *Anaerovoracaceae* (ASVs 6, 7, and 8) (formerly *Clostridiales* family XIII (Parks et al., 2018)), and members of the genus *Caproiciproducens* (ASVs 10 and 29) related to *Caproicibacter fermentans* (Supplementary Table 4). Although the mechanism through which caproate was produced in the presence of H_2_ remains unknown, correlation analysis may provide some hints.

In communities B and C, no bacterial taxon correlated to H_2_ consumption rates could be found. This was likely due to the more intense consumption of H_2_ for methanogenesis in inhibitor-free cultures of these communities. Considering that no culture in this study showed net H_2_ formation, negative correlations to H_2_ consumption rate to certain bacterial taxa (as found in Supplementary Figures 8, 9) may simply mean that bacteria were slower H_2_ consumers than archaea. Still, abundances of ASVs assigned to *Clostridium* sensu stricto 12 correlated positively to H_2_ consumption in community A (Supplementary Figure 7), whereas other ASVs affiliated to *Clostridium* sensu stricto 12 were linked to caproate formation (Supplementary Figures 8, 9). With relatively high abundances in all communities, the genus *Clostridium* sensu stricto 12 thrived particularly in communities A and C after lactate and ethanol addition (Figure 8). So far, hydrogenotrophy and caproate production have not been reported as functions of *C. luticellarii* (Wang et al., 2015). However, its genome harbors typical genes of the Wood-Ljungdahl pathway (Poehlein et al., 2018) and *C. luticellarii* was the main candidate to elongate propionate to valerate and to produce *i*-butyrate in a recent study (de Smit et al., 2019). Moreover, a caproate producer recently isolated in our laboratory, Clostridiales bacterium isolate BL-3, is also closely related to *C. luticellarii* (Liu et al., 2020b,c). *C. luticellarii* is closely related to *C. ljungdahlii* (a solventogen and syngas fermenter) and *C. kluyveri* (a chain elongator) (Wang et al., 2015; de Smit et al., 2019). Moreover, it is important to highlight that the fact that no ethanol accumulated in our experiments should not exclude the possibility of this ED being the one intermediating the H_2_-to-caproate through interspecies transfer. With ethanol having faster consumption kinetics than those of H_2_ in acidogenic cultures (González-Cabaleiro et al., 2013; Weimer et al., 2015), intermediate ethanol may be present in concentrations below the detection limit or its production-consumption cycles could have been overlooked with the sampling frequency of this study. In fact, sporadic occurrence of small ethanol concentrations (12 - 130 mg/L, data not shown) was observed. However, with the experimental design and methodology adopted here, no conclusions could be drawn from this observation.

Judging by their notable abundances in different experiments, ASVs assigned to the *Anaerovoracaceae* fared well under a broad range of conditions and substrates (Figure 8), including H_2_, lactate, and ethanol, besides being one of the suspects to produce caproate in community B. Such versatility is seen in only few caproate producers, being *E. limosum* one of the few examples, albeit it does not grow with ethanol (Wade, 2015). For reference, the distantly related *Eubacterium pyruvativorans* (Supplementary Table 4) is a caproate producer that does not use ethanol, grows slowly on lactate and needs SCC to realize a CE metabolism that is uninhibited by high H_2_ partial pressure (Wallace et al., 2004).

The ASVs related to *Caproiciproducens* (in all communities) and *Megasphaera* (in community C) had minor but consistent abundances along the enrichment (Figure 8). ASVs of these two genera had established presence before lactate addition, hence their growth could have relied on the presence of yeast extract, interspecies metabolite transfer or H_2_ consumption. Their closest related species (*Caproicibacter fermentans* and *M. elsdenii*) are best known for their abilities to produce caproate from sugars or lactate (*M. elsdenii*) (Rosenberg et al., 2014; Flaiz et al., 2019; Lee et al., 2020), but neither for hydrogenotrophy nor ethanol consumption. Interestingly, in one of the first reports on *M. elsdenii* (Elsden and Lewis, 1953), the species was shown to consume H_2_ together with pyruvate while realizing CE metabolism when SCC were available. For instance, *Caproiciproducens galactitolivorans*, a chain elongator closely related to *Caproicibacter fermentans*, has not been observed to utilize ethanol but was reported to have its growth enhanced in co-culture with other ethanol-, acetate-, and butyrate-producing bacteria (Kim et al., 2015).

## Conclusion

Overall, the simultaneous occurrence of phylogenetically distinct families within the *Firmicutes* (i.e., *Clostridiaceae*, *Veillonellaceae*, *Ruminococcaceae*, and *Anaerovoracaceae*) hints to a broad taxonomic range of suspected caproate producers that thrive in the presence of H_2_. Our results suggest a widespread effect of the synergy between H_2_, lactate, and ethanol on caproate production by complex communities. This study adds up to the growing body of evidence that abundant H_2_ availability can improve efficiency of MCC-producing microbiota ultimately acting as a co-electron donor. Still, plenty remains to be understood regarding the underlying mechanisms through which this synergy occurs. For that, we suggest designing studies that better resolve the metabolic network in such complex microbiota with the help of, for instance, meta-omics approaches.

The different conditions tested in batch cultures can serve as a starting point to better devise strategies that alleviate ED scarcity on continuous CE reactors with the help of H_2_. Instead of depending exclusively on a conventional ED or on H_2_, we advise to develop a CE process in which both types of EDs are co-fed in order to improve MCC production rates, concentrations, and selectivities. For kick-starting the bioreactor, the more diverse inoculum should be favored over the more specialized one. When methanogenesis misroutes electrons from H_2_, ethylene should be favored over 2-BES as an inhibitor in order to not collaterally inhibit *i-*butyrate formation.

## Data Availability Statement

The datasets generated for this study can be found in the EMBL European Nucleotide Archive (ENA) under accession number PRJEB40259 (http://www.ebi.ac.uk/ena/data/view/PRJEB40259).

## Author Contributions

FB, SK, and HS: conceptualization, methodology, and writing (review & editing). FB: investigation, formal analysis, data curation, visualization, and writing (original draft preparation). SK and HS: supervision and project administration. All authors have read and agreed to the published version of the manuscript.

## Conflict of Interest

The authors declare that the research was conducted in the absence of any commercial or financial relationships that could be construed as a potential conflict of interest.

## Footnotes

1 http://www.ebi.ac.uk/ena/data/view/PRJEB40259
