# Supplementary material for: Hydrogen as a Co-electron Donor for Chain Elongation With Complex Communities
Source: Front Bioeng Biotechnol. 2021 Mar 31;9:650631. doi: 10.3389/fbioe.2021.650631 (PMC8059637; doi:10.3389/fbioe.2021.650631)
Supplement: Supplementary file 1 [file Data_Sheet_1.PDF]

Supplementary Material

1 Supplementary Figures and Tables

1.1 Supplementary Tables

Supplementary Table 1. Duration of each experiment in days.

Experiment	Community A	Community B	Community C
Acidic pH values	51	-	21
Acetate concentration	63	92	63
Acidic/neutral pH or methanogenesis inhibition	77	48	77
Lactate and ethanol addition	26	40	26

Supplementary Table 2. Components and converting factors for electron balances.

Compound (formula)	Molar mass (g mol^{-1})	mol e^-/mol
Biomass and yeast extract ($C_1H_{1.8}O_{0.5}N_{0.2}$)	24.6	4.2
Acetic acid/acetate ($C_2H_4O_2$)	60.0	8.0
Ethanol (C_2H_6O)	46.0	12.0
Lactic acid/lactate ($C_3H_6O_3$)	90.0	12.0
Butyric acid/butyrate ($C_4H_8O_2$)	88.0	20.0
Caproic acid/caproate ($C_6H_{12}O_2$)	116.1	32.0
Propionic acid/propionate ($C_3H_6O_2$)	74.0	14.0
Valeric acid/valerate ($C_5H_{10}O_2$)	102.1	26.0
i-Butyric acid/i-butyrate ($C_4H_8O_2$)	88.0	20.0
H_2	2.0	2.0
CO_2	44.0	0.0
CH_4	16.0	8.0

Supplementary Table 3. Production rates of butyrate and caproate, in mg L^{-1} d^{-1}. Errors are standard errors.

Experiment	Condition	Community A				Community B				Community C			
		Butyrate		Caproate		Butyrate		Caproate		Butyrate		Caproate	
		H_2-free	w/ H_2	H_2-free	w/ H_2	H_2-free	w/ H_2	H_2-free	w/ H_2	H_2-free	w/ H_2	H_2-free	w/ H_2
Acidic pH values	pH 4.8	9.7 ± 0.5	11 ± 2	11 ± 1	9.5 ± 0.6	-	-	-	-	5.0 ± 0.2	13.5 ± 0.2	1.2 ± 0.8	5 ± 1
	pH 5.5	46 ± 3	57 ± 2	11 ± 2	12.8 ± 0.1	-	-	-	-	3 ± 2	1.1 ± 0.2	3.3 ± 0.9	4 ± 1
Acetate concentration	Acetate 100 mM	0.2 ± 0.9	16 ± 5	-0.8 ± 0.1	-0.4 ± 0.1	1.0 ± 0.4	5 ± 2	5.6 ± 0.7	6.2 ± 0.4	1.9 ± 0.3	8 ± 2	0.1 ± 0.2	2 ± 1
	Acetate 200 mM	0 ± 1	12 ± 2	-0.1 ± 0.4	-0.4 ± 0.1	3.0 ± 0.1	12.0 ± 0.2	4.6 ± 0.1	5.8 ± 0.7	2.7 ± 0.2	14 ± 4	0.3 ± 0.4	3.9 ± 0.8
Acidic/neutral pH or methanogenesis inhibition	pH 5.5 or uninhibited	0.3 ± 0.2	19 ± 9	0.0 ± 0.0	0.0 ± 0.0	4.5 ± 0.0	23 ± 1	4.2 ± 0.1	25.7 ± 0.7	1.2 ± 0.4	12 ± 8	0.7 ± 0.3	4 ± 2
	pH 7.0 or inhibited	0.3 ± 0.1	-0.5 ± 0.1	0.0 ± 0.0	0.0 ± 0.0	4.3 ± 0.2	32 ± 3	4 ± 1	14 ± 6	2 ± 1	30 ± 17	2 ± 1	10 ± 3
Lactate and ethanol addition	pH 5.5 or uninhibited	181 ± 8	317 ± 107	0.0 ± 0.0	8 ± 10	44.4 ± 0.7	49 ± 22	34 ± 1	86 ± 17	116.2 ± 0.6	190 ± 12	76.2 ± 0.4	72 ± 2
	pH 7.0 or inhibited	35	172.9 ± 0.7	0	0.0 ± 0.0	49 ± 8	32 ± 39	33 ± 4	61.0 ± 0.5	116.1 ± 0.3	167	43 ± 10	107

Supplementary Table 4. Taxonomy table with the 30 most abundant ASVs in the dataset with family and genus assigned according to the Silva 138 database. The closest cultured species is shown together with its BLAST similarity and the NCBI accession number. *ASV_0017 also has 100% similarity with an isolate from reactor A (Liu et al., 2020a; Liu et al., 2020b), i.e. Clostridiales bacterium isolate BL-6 (LR778135.1).

	Family	Genus	Most similar cultured species (BLAST similarity, accession number)
ASV_0001	Clostridiaceae	Clostridium sensu stricto 12	*Clostridium tyrobutyricum* (100%, NR_044718.2)
ASV_0002	Methanobacteriaceae	Methanobacterium	*Methanobacterium congolense* (98.7%, NR_028175.1)
ASV_0003	Clostridiaceae	Clostridium sensu stricto 12	*Clostridium luticellarii* (98.3%, NR_145907.1)
ASV_0004	Methanobacteriaceae	Methanobacterium	*Methanobacterium congolense* (98.2%, NR_028175.1)
ASV_0005	Clostridiaceae	Clostridium sensu stricto 12	*Clostridium luticellarii* (99.0%, NR_145907.1)
ASV_0006	Anaerovoracaceae	-	*Eubacterium pyruvativorans* (92.0%, NR_042074.1)
ASV_0007	Anaerovoracaceae	-	*Eubacterium pyruvativorans* (91.8%, NR_042074.1)
ASV_0008	Anaerovoracaceae	-	*Eubacterium pyruvativorans* (92.1%, NR_042074.1)
ASV_0009	Clostridiaceae	Clostridium sensu stricto 12	*Clostridium luticellarii* (99.8%, NR_145907.1)
ASV_0010	Ruminococcaceae	Caproiciproducens	*Caprobacter fermentans* (95.0%, MN851263.1)
ASV_0011	Methanobacteriaceae	Methanobrevibacter	*Methanobrevibacter boviskoreani* (100%, NR_118565.1)
ASV_0012	Atopobiaceae	Olsenella	*Olsenella scatoligenes* (100%, NR_134781.1)
ASV_0016	Lactobacillaceae	Lactobacillus	*Lactobacillus mucosae* (100%, MT613566.1)
ASV_0017*	Ruminococcaceae	Caproiciproducens	*[Clostridium] sporosphaeroides* (96.8%, NR_044835.2)
ASV_0018	Peptostreptococcales-Tissierellales	-	*Soehngenia saccharolytica* (92.8%, NR_117382.1)
ASV_0020	Oscillospiraceae	Oscillibacter	*Oscillibacter ruminantium* (92.1%, NR_118156.1)
ASV_0021	Clostridiaceae	Clostridium sensu stricto 12	*Clostridium luticellarii* (97.8%, NR_145907.1)
ASV_0022	Limnochordia class	-	*Natranaerobaculum magadiense* (88.8%, NR_135713.1)
ASV_0029	Ruminococcaceae	Caproiciproducens	*Caprobacter fermentans* (95.0%, MN851263.1)
ASV_0033	Veillonellaceae	Megasphaera	*Megasphaera hexanoica* (100%, NR_157635.1)
ASV_0035	Actinomycetaceae	Actinomyces	*Actinomyces polynesiensis* (100%, NR_144691.1)
ASV_0036	Lactobacillaceae	Lactobacillus	*Lactobacillus mucosae* (100%, MT539050.1)
ASV_0038	Erysipelotrichaceae	Solobacterium	*Anaerorhabdus furcosa* (90.8%, NR_117779.1)
ASV_0039	Acidaminococcaceae	Acidaminococcus	*Acidaminococcus fermentans* (96.3%, NR_037018.1)
ASV_0042	Lachnospiraceae	Syntrophococcus	*[Eubacterium] cellulosolvens* (95.2%, NR_026106.1)
ASV_0043	Limnochordia class	-	*Sporanaerobacter acetigenes* (87.8%, NR_117381.1)
ASV_0048	Veillonellaceae	Megasphaera	*Megasphaera elsdenii* (99.8%, NR_113306.1)
ASV_0051	Peptostreptococcales-Tissierellales	-	*Soehngenia saccharolytica* (93.0%, NR_117382.1)
ASV_0054	Propionibacteriaceae	Cutibacterium	*Cutibacterium avidum* (99.5%, NR_118647.1)
ASV_0073	Coriobacteriales Incertae Sedis	-	*Gordonibacter pamelaeae* (91.1%, MK544834.1)

1.2 Supplementary Figures

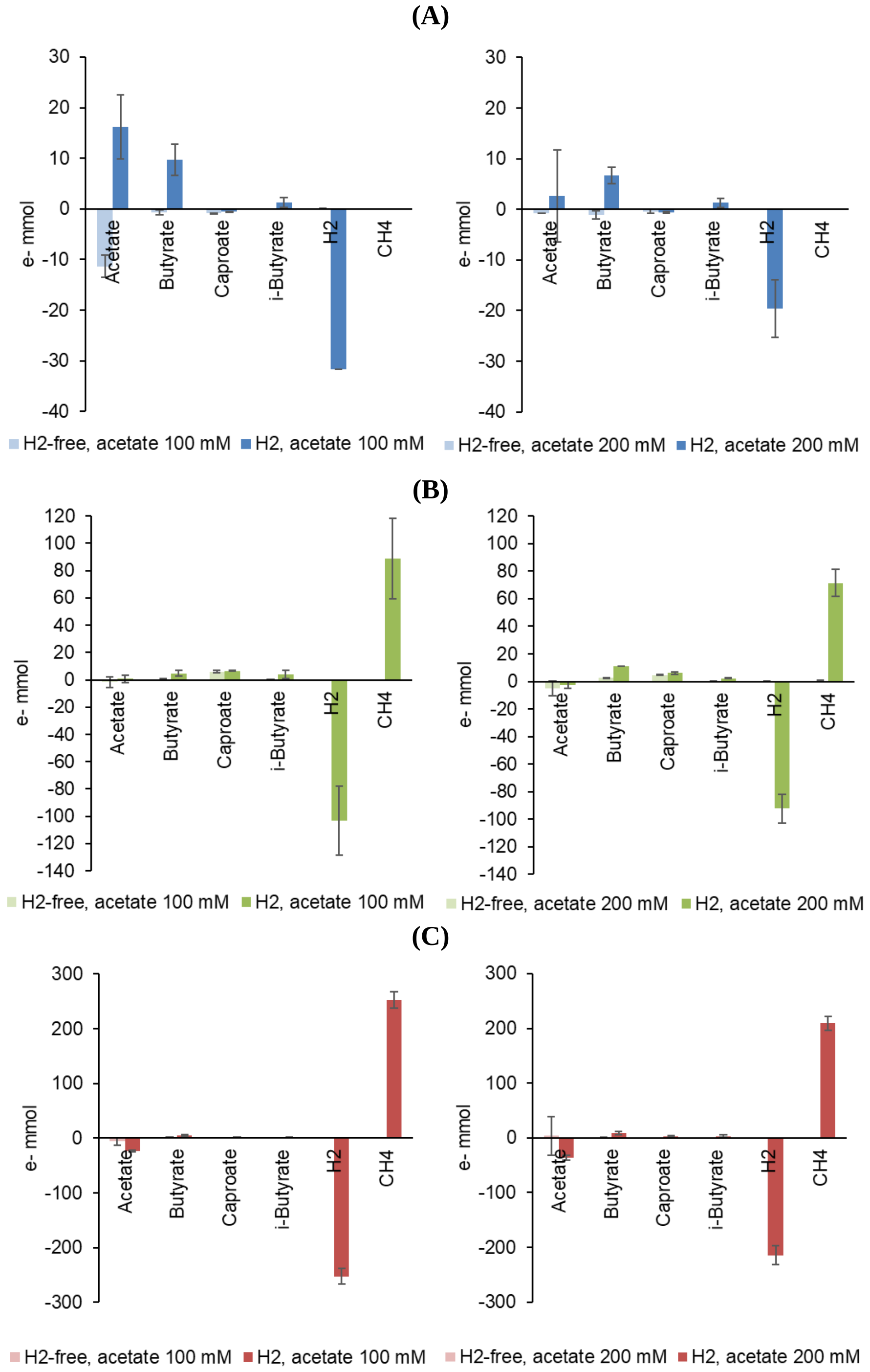

Supplementary Figure 1. Electron balances for the acetate concentration experiment with communities A (A), B (B), and C (C) in equivalent mmols of electrons. Biomass, lactate, propionate, and valerate are not shown due to negligible variations for these components. Error bars are standard error.

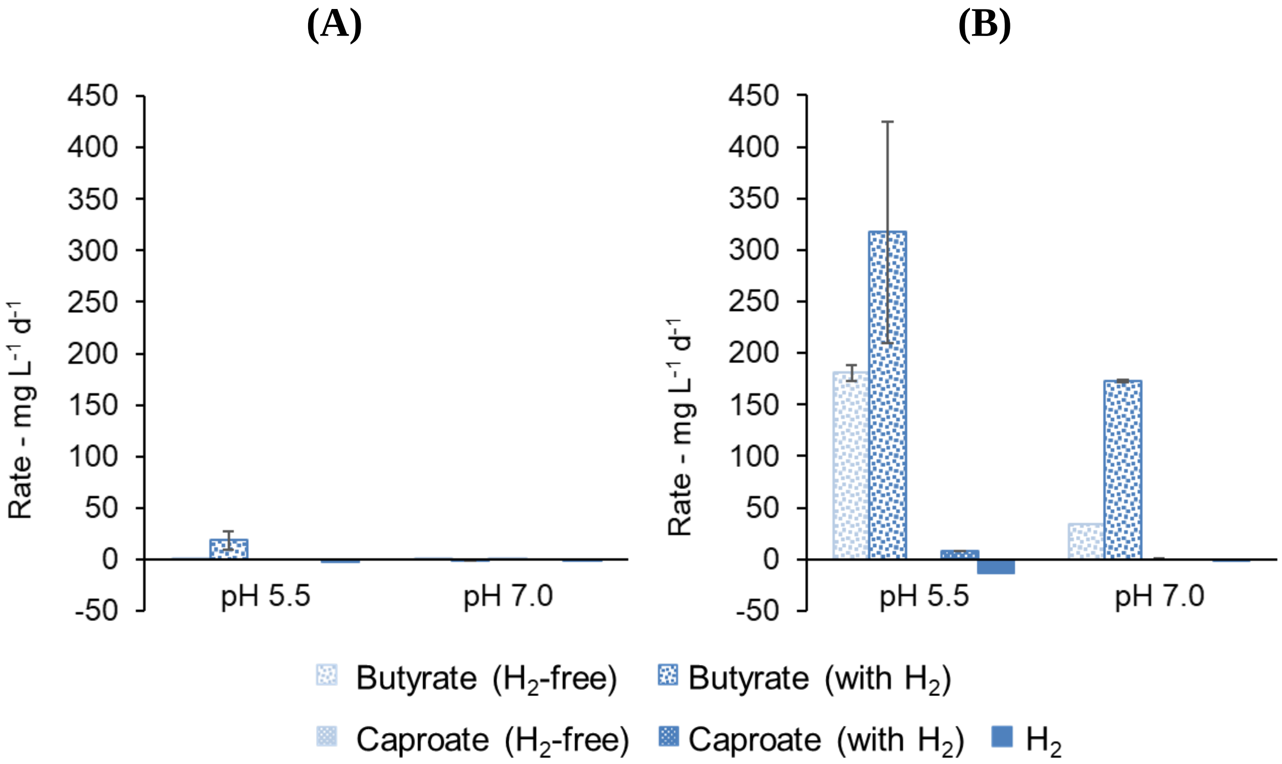

Supplementary Figure 2. Effects of pH and H_2 before (A) and after (B) ethanol and lactate addition on rates of caproate, butyrate, and H_2 by community A. One of the duplicates of community A without H_2 at pH 7.0 in (B) was lost and no standard error bar is shown for this condition. Error bars are standard errors.

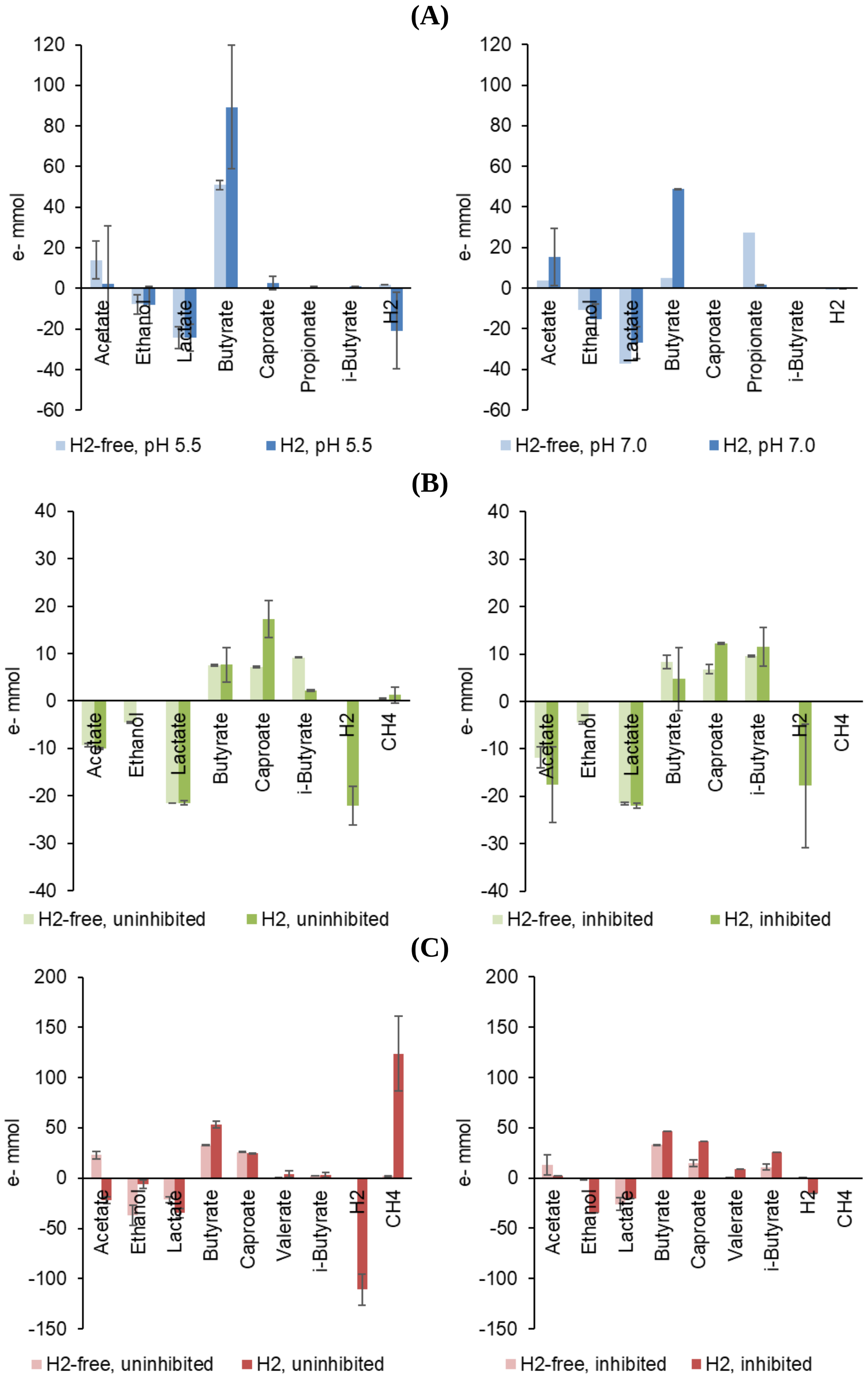

Supplementary Figure 3. Electron balances for the lactate and ethanol addition experiment for communities A (A), B (B), and C (C) in equivalent mmols of electrons. Components that had negligible variation throughout the experiment are omitted. Error bars are standard error.

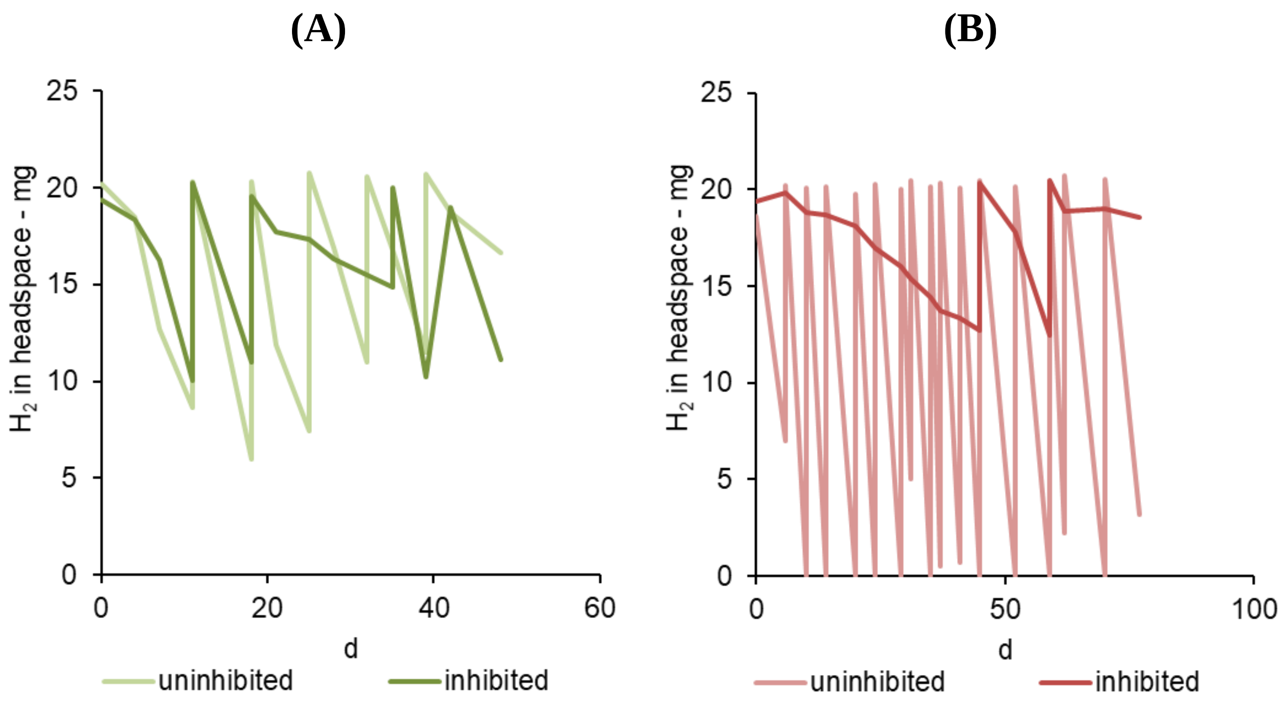

Supplementary Figure 4. Amount of H_2 in the headspace for communities B (A) and C (B) in the methanogenesis inhibition experiment. In community B neither inhibited nor uninhibited bottles had complete H_2 depletion. On the other hand, uninhibited culture bottles with community C faced H_2 depletion often.

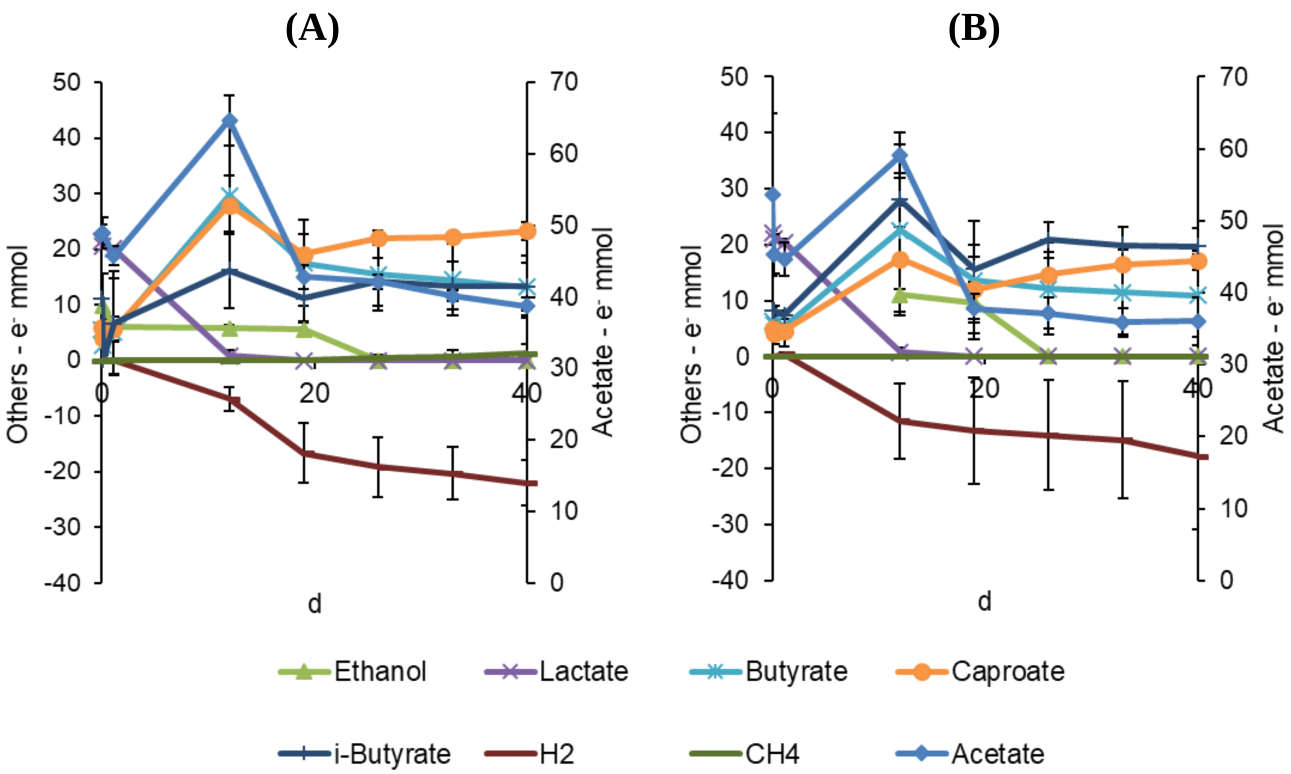

Supplementary Figure 5. Kinetics profiles for community B after lactate and ethanol addition to the bottles without (A) and with (B) methanogenesis inhibitor. Error bars are standard error.

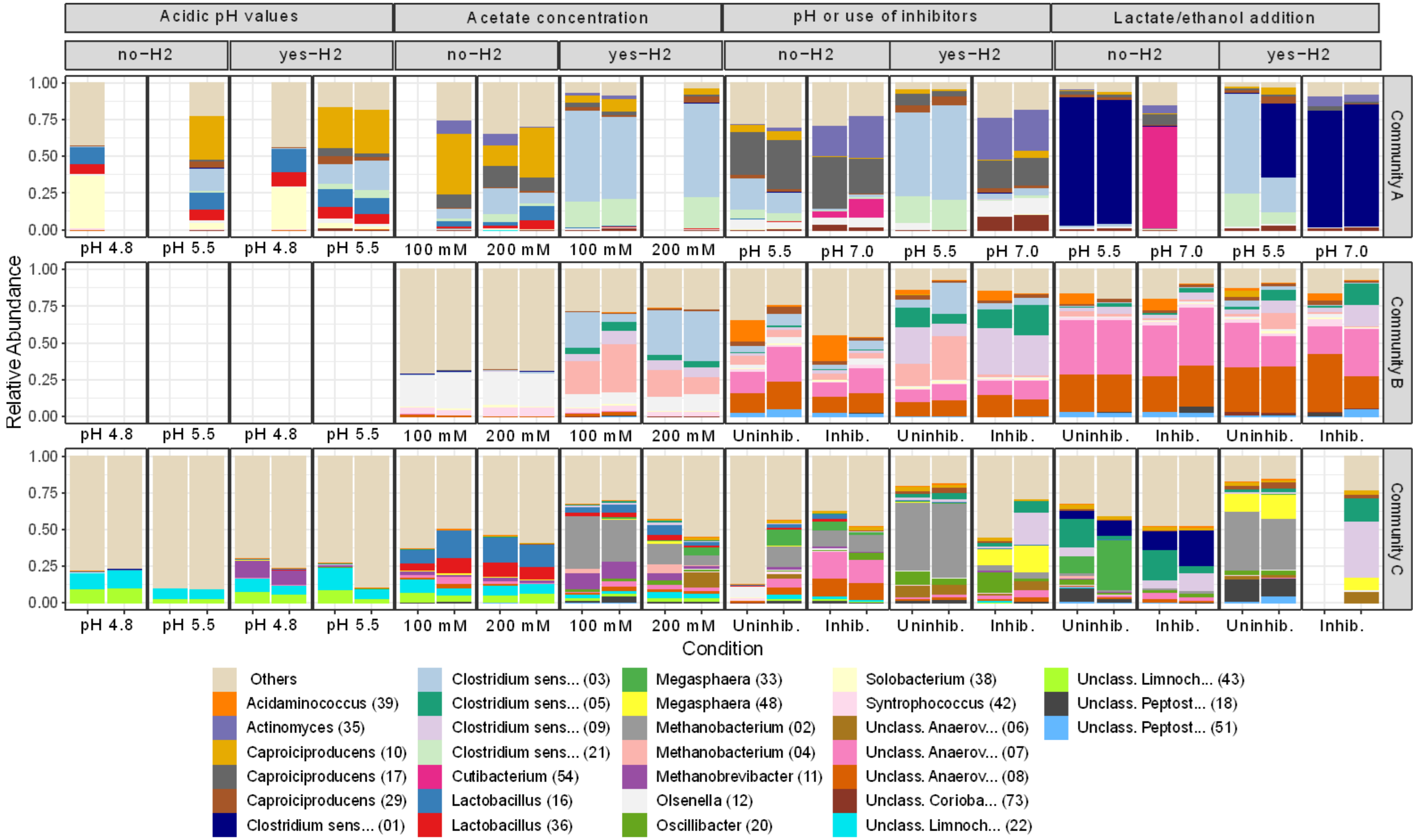

Supplementary Figure 6. Community profiles at each condition tested in the study resolved to the ASV level for the 30 most abundant ASVs in the dataset (ASV number in parenthesis). Duplicates are shown. Slots left blank represent samples that could not be sequenced.

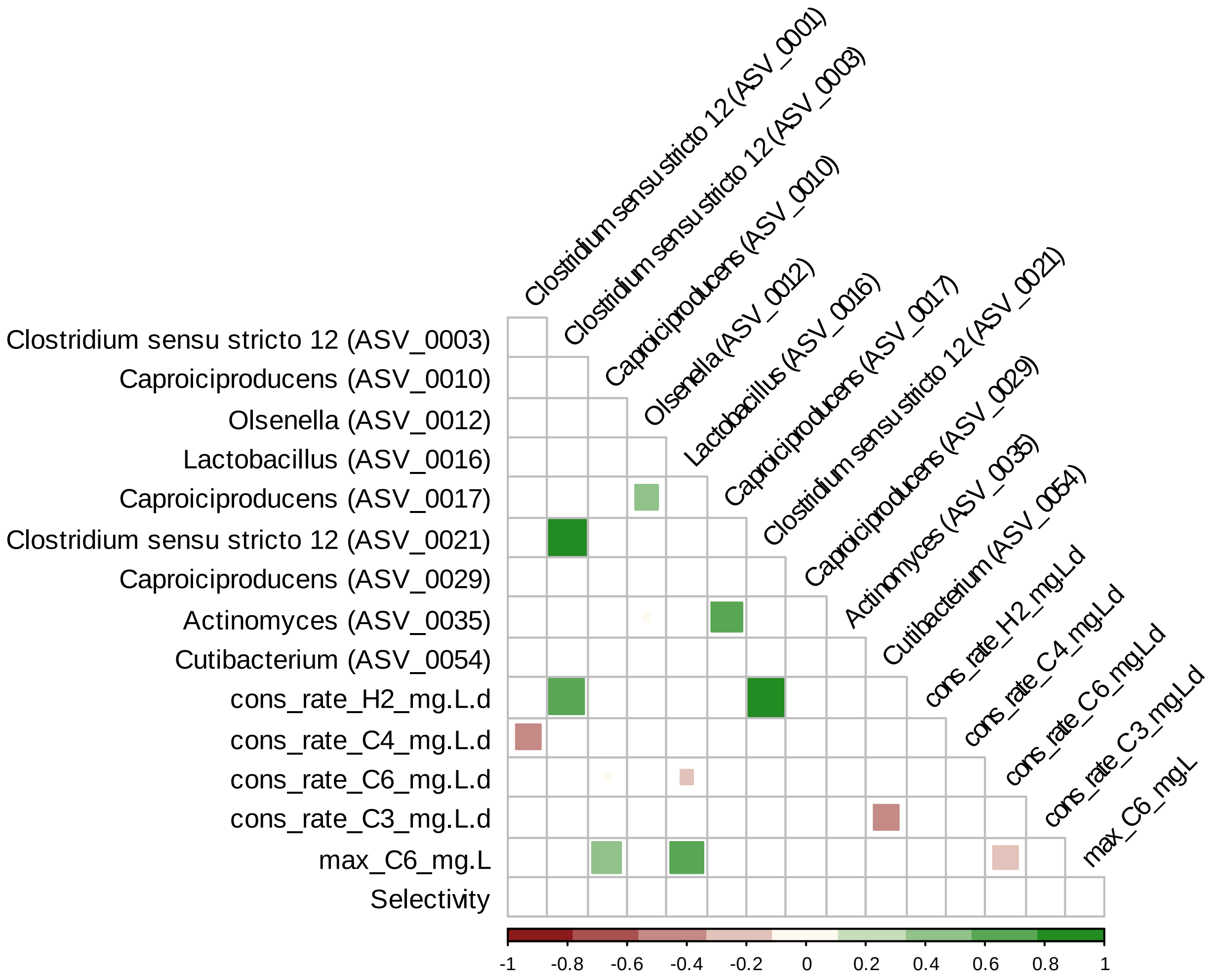

Supplementary Figure 7. Spearman correlations ($p<0.01$) between the 10 most abundant ASVs of community A and abiotic parameters (rates of H_2, butyrate, caproate, and propionate, as well as maximum caproate concentration, and selectivity of EDs to caproate). As a convention, all rates are presented as consumption rates. Thus, a negative correlation to caproate consumption rate is equivalent to a positive correlation to caproate formation rate and vice-versa.

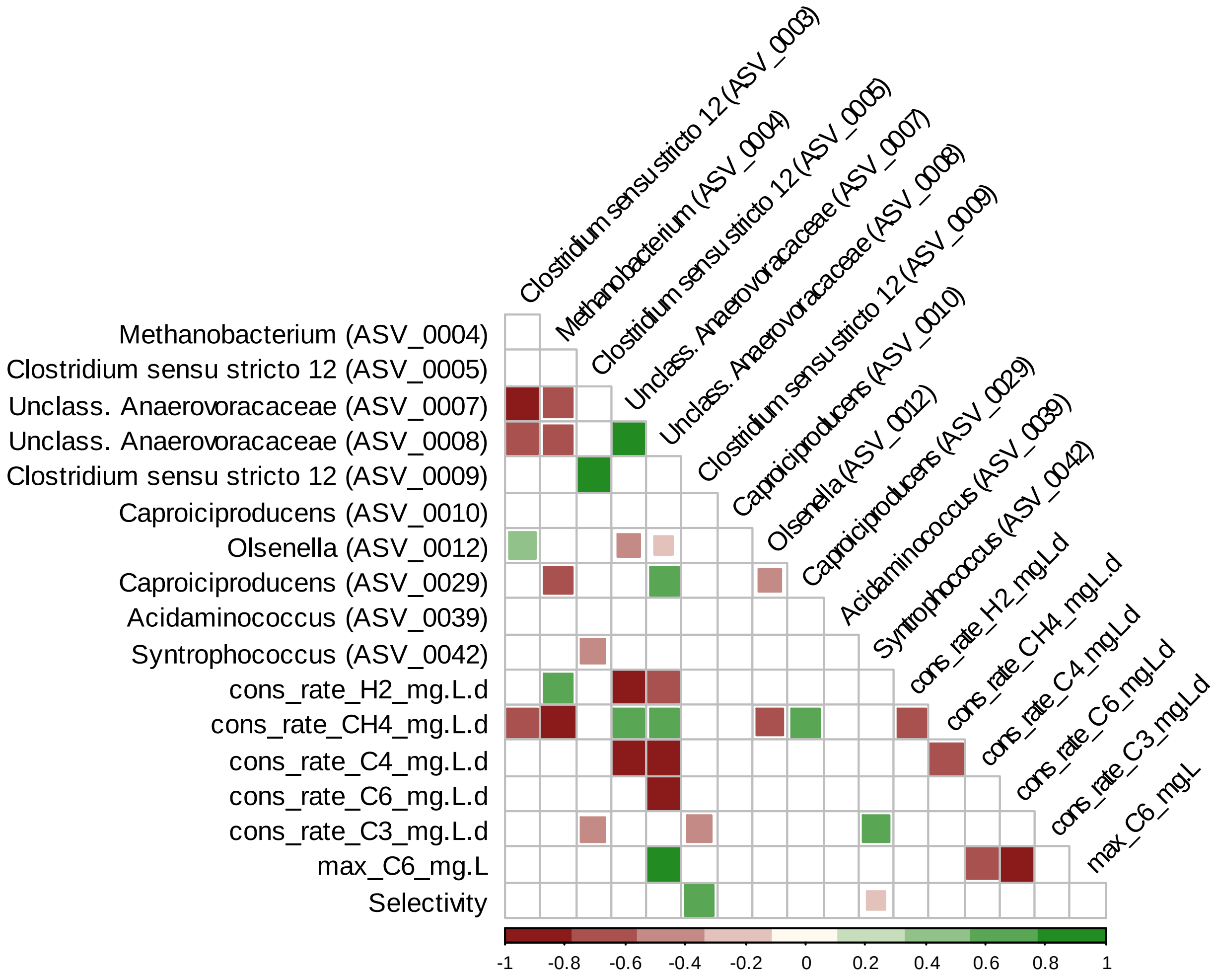

Supplementary Figure 8. Spearman correlations ($p<0.01$) between the 10 most abundant ASVs of community B, ASV 10, and abiotic parameters (rates of H_2, CH_4, butyrate, caproate, and propionate, as well as maximum caproate concentration, and selectivity of EDs to caproate). As a convention, all rates are presented as consumption rates. Thus, a negative correlation to caproate consumption rate is equivalent to a positive correlation to caproate formation rate.

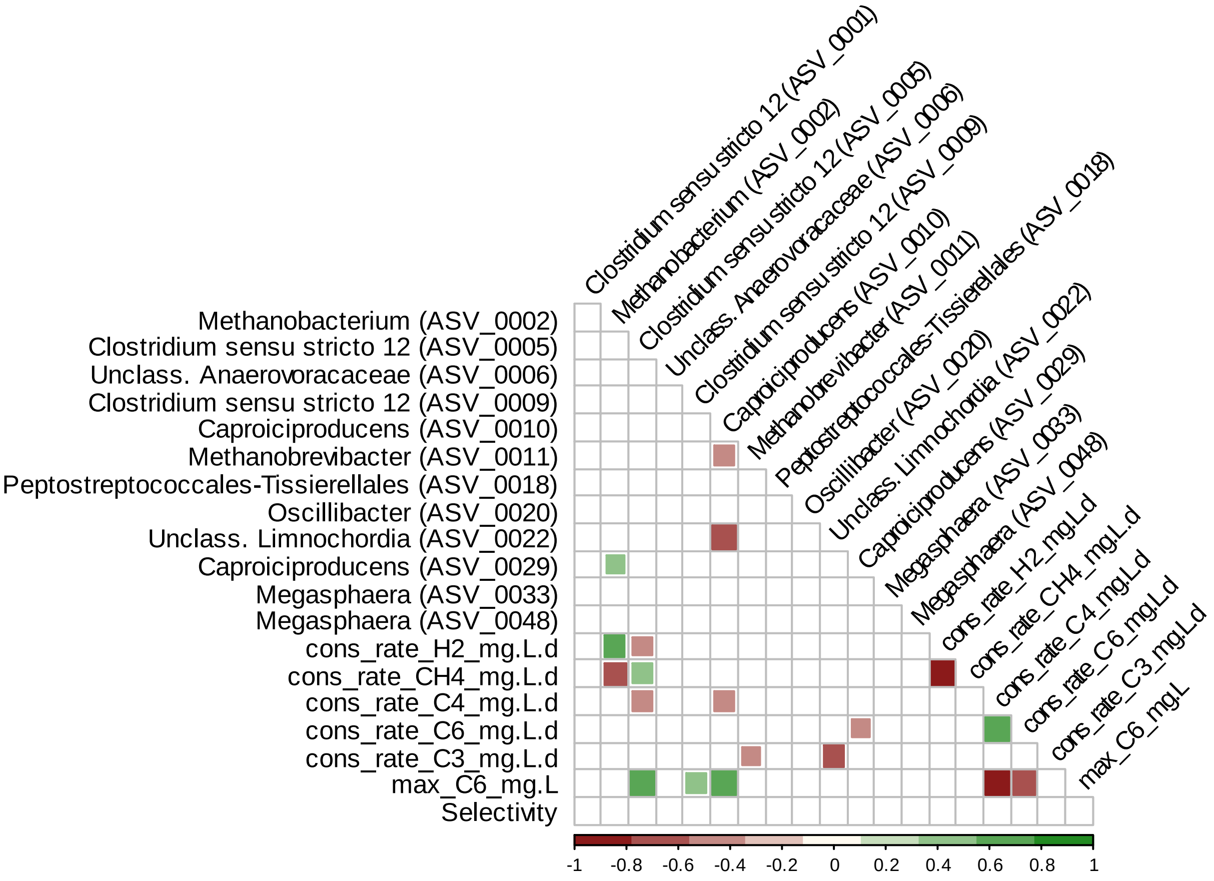

Supplementary Figure 9. Spearman correlations ($p<0.01$) between the 10 most abundant ASVs of community C, ASV 6, ASV 10, ASV 29 and abiotic parameters (rates of H_2, CH_4, butyrate, caproate, and propionate, as well as maximum caproate concentration, and selectivity of EDs to caproate). As a convention, all rates are presented as consumption rates. Thus, a negative correlation to caproate consumption rate is equivalent to a positive correlation to caproate formation rate.

2 References

Liu, B., Popp, D., Muller, N., Strauber, H., Harms, H., and Kleinsteuber, S. (2020a). Three Novel Clostridia Isolates Produce n-Caproate and iso-Butyrate from Lactate: Comparative Genomics of Chain-Elongating Bacteria. *Microorganisms* 8(12). doi: 10.3390/microorganisms8121970.

Liu, B., Popp, D., Strauber, H., Harms, H., and Kleinsteuber, S. (2020b). Draft Genome Sequences of Three Clostridia Isolates Involved in Lactate-Based Chain Elongation. *Microbiol Resour Announc* 9(32). doi: 10.1128/MRA.00679-20.
